# Single-cell resolution analysis reveals the preparation for reprogramming the fate of stem cell niche in cotton lateral meristem

**DOI:** 10.1186/s13059-023-03032-6

**Published:** 2023-08-25

**Authors:** Xiangqian Zhu, Zhongping Xu, Guanying Wang, Yulong Cong, Lu Yu, Ruoyu Jia, Yuan Qin, Guangyu Zhang, Bo Li, Daojun Yuan, Lili Tu, Xiyan Yang, Keith Lindsey, Xianlong Zhang, Shuangxia Jin

**Affiliations:** 1 Hubei Hongshan Laboratory, National Key Laboratory of Crop Genetic Improvement, Huazhong Agricultural University, Wuhan, 430070 Hubei China; 2 Xinjiang Key Laboratory of Crop Biotechnology, Institute of Nuclear and Biological Technology, Xinjiang Academy of Agricultural Sciences, Wulumuqi, 830000, Xinjiang China; 3 Department of Biosciences, Durham University, Durham, DH1 3LE UK

**Keywords:** Cotton, Plant regeneration, scRNA-seq, Gene regulatory network, Gene functional verification

## Abstract

**Background:**

Somatic embryogenesis is a major process for plant regeneration. However, cell communication and the gene regulatory network responsible for cell reprogramming during somatic embryogenesis are still largely unclear. Recent advances in single-cell technologies enable us to explore the mechanism of plant regeneration at single-cell resolution.

**Results:**

We generate a high-resolution single-cell transcriptomic landscape of hypocotyl tissue from the highly regenerable cotton genotype Jin668 and the recalcitrant TM-1. We identify nine putative cell clusters and 23 cluster-specific marker genes for both cultivars. We find that the primary vascular cell is the major cell type that undergoes cell fate transition in response to external stimulation. Further developmental trajectory and gene regulatory network analysis of these cell clusters reveals that a total of 41 hormone response-related genes, including *LAX2*, *LAX1*, and *LOX3*, exhibit different expression patterns in the primary xylem and cambium region of Jin668 and TM-1. We also identify novel genes, including *CSEF*, *PIS1*, *AFB2*, *ATHB2*, *PLC2*, and *PLT3*, that are involved in regeneration. We demonstrate that *LAX2*, *LAX1* and *LOX3* play important roles in callus proliferation and plant regeneration by CRISPR/Cas9 editing and overexpression assay.

**Conclusions:**

This study provides novel insights on the role of the regulatory network in cell fate transition and reprogramming during plant regeneration driven by somatic embryogenesis.

**Supplementary Information:**

The online version contains supplementary material available at 10.1186/s13059-023-03032-6.

## Background

Plant somatic cell regeneration includes organogenesis and somatic embryogenesis pathways, which represent forms of fertilization-independent development. Plant somatic embryogenesis is a process in which somatic cells of a variety of tissues are cultured in vitro to undergo de-differentiation and then re-differentiation into somatic embryos and can develop into whole plants [1]. In plants, callus formation is a key step in the cell reprogramming pathway for somatic embryogenesis and is triggered and influenced by many factors, including wounding and hormonal (auxin, cytokinin, abscisic acid, and ethylene) status of the tissue. The remarkable totipotency of plant somatic cells [2] has been exploited for the development of methodologies for genetic transformation and plant regeneration used in the biotechnology and molecular breeding of major crops. However, there is still limited information on whether somatic embryos originate from single or multiple cells in explant tissues and the mechanisms controlling cell reprogramming to initiate embryonic development.

Cotton is an attractive model for studying somatic embryogenesis because it produces both embryogenic callus (EC) and non-embryogenic callus (NEC), which provide useful material for molecular analysis. The main factor affecting somatic embryogenesis in cotton is the genotype of the explant chosen for tissue culture [3]. Currently, only very few genotypes, such as Coker 201/310/312/315 [4–6], ZM24 [7], Simian 3 [8], YZ1 [9], and Jin668 [6], exhibit high regeneration efficiency via somatic embryogenesis. Among these, Jin668 is a newly developed elite genotype and widely used in genetic engineering and genome editing [2, 6, 8, 10–16]. However, the mechanism underlying its strong regeneration ability is not understood. In recent years, the morphological and molecular mechanisms of somatic embryogenesis have been emerging as an exciting new focus of research, and transcription factors (TFs) are considered to play a major regulatory role. Ectopic expression of certain key TFs (associated with exogenous hormones, chromatin remodeling and response to stress conditions [17]) has been used to understand and improve plant regeneration efficiency [18]. These include *BABY BOOM* (*BBM*) [19], EMBRYOMAKER (*EMK*) [20], SOMATIC EMBRYO RELATED FACTOR 1 (*SERF1*) [21], *WOUND INDUCED DEDIFFERENTIATION1* (*WIND1*), *WIND2*, *WIND3*, *WIND4* belonging to AP2/ERF family, *WUSCHEL2* (*WUS2*) [19], *AGAMOUS-LIKE15* (*AGL15*) [22], LEAFY COTYLEDON1 (*LEC1*) [23] and *LEC2* [24], FUSCA3 (*FUS3*), GROWTH-REGULATING FACTOR-INTERACTING FACTOR (*GRF*-*GIF*) [25], SHOOT MERISTEMLESS (*STM*), KNOTTED1 (*KN1*), ENHANCER OF SHOOT REGENERATION (*ESR1* and *ESR2*), and MONOPTEROS (*MP*). Besides TFs, other protein-coding genes [26] and epigenetic regulation [27–29] also regulate the reprogramming of somatic cells. In cotton, *GhHmgB3* [30], *GhCKI* [31], *GhL1L1* [32], and *GhTCE1* [33] regulate auxin concentrations during the dedifferentiation process. However, many details of gene regulation during somatic embryogenesis and genotype-dependent regeneration in cotton remain unknown.

Regeneration from callus involves the specification of stem cells, and different explant cell types have different regeneration abilities [13, 34, 35]. For example, pericycle cells of alfalfa can produce lateral roots [36] and most callus in *Arabidopsis* is derived from dividing cells with a pericycle-like gene expression profile [37]. The embryogenic cells of carrot root are derived from all primary tissues of the stele, i.e., pericycle, phloem, and xylem [2, 38]. Morphological studies also support the connection between somatic embryogenesis and procambium or primary vascular tissue [15, 16, 39, 40]. In the tissue culture of *Arabidopsis thaliana*, cells capable of embryogenesis were found to be mainly derived from procambium cells which highly express the *SOMATIC EMBRYOGENESIS RECEPTOR KINASE 1* (*SERK1*) gene [41]. Our previous research also revealed that during *Agrobacterium*-mediated transformation using cotton hypocotyl segments as explants, the *Agrobacterium* mainly infected the primary vascular tissue and then stably transformed callus prior to regeneration [10]. Despite the high regeneration potential of plant cells, spatial and temporal restriction of this process is also found to be highly correlated with the location of stem cells, meristematic cells, or highly reprogrammable cells. Less differentiated cell types, such as vascular cambium cells, may be one of major reprogrammable cell types. Therefore, precise analysis of the process of somatic cell reprogramming at the single-cell level is of great potential value for improving our understanding of the regulatory mechanism of plant regeneration.

Recent advances in single-cell RNA sequencing (scRNA-seq) provide an unprecedented opportunity to systematically identify the genes expressed, and the regulatory network underpinning somatic embryogenesis, at the single-cell level [42–44]. The single-cell landscape of the *Arabidopsis* root reveals highly heterogeneous, distinct patterns of ion assimilation and hormonal responses in different cell clusters [42], revealing the key developmental regulatory factors that promote the transformation of stem cells into different cell types [45] and has identified cell type-specific genes and regulatory patterns of TFs during root development [46]. Likewise, scRNA-seq analysis of the shoot apical meristem (SAM) of maize uncovered evolutionarily divergent and conserved signatures of plant shoot meristems [43]. Comparative analysis of root scRNA-seq data from rice and *Arabidopsis* showed that the transcriptome of most cell types was quite different and indicates the importance of performing single-cell analysis for species-specific tissues [44]. Combined with scRNA-seq and ATAC-seq, *WUS* and *DRN* were found to be essential for the regeneration of *Arabidopsis* mesophyll cells [47]. scRNA-seq of *Arabidopsis* callus also found that the ratio of auxin to cytokinin, and the sensitivity of cytokinin in the middle layer cells of callus, is necessary for the acquisition of pluripotency [48].

We recently applied scRNA-seq technology to cotton fiber cell development [49]. To explore the transcriptional dynamics of cotton hypocotyl primary vascular tissue during tissue culture for somatic embryogenesis, scRNA-seq was performed at 0, 1, 6, and 12 h after callus induction (HACI) using two cotton cultivars, TM-1 (recalcitrant genotype) and Jin668 (highly-regenerable). CRISPR/Cas9 genome editing and transgenic overexpression were used to investigate the potential roles of *LAX2*, *LAX1*, and *LOX3* genes identified by scRNA-seq. This study provides novel insight into the transcriptomic landscape of different cell types of cotton hypocotyl at single-cell resolution and identifies candidate genes involved in the control plant regeneration via somatic embryogenesis. This offers potential opportunities for improving the efficiency of regeneration in currently recalcitrant genotypes.

## Results

### Construction of single-cell transcription landscape of cotton hypocotyl during dedifferentiation

**Fig. 1:**
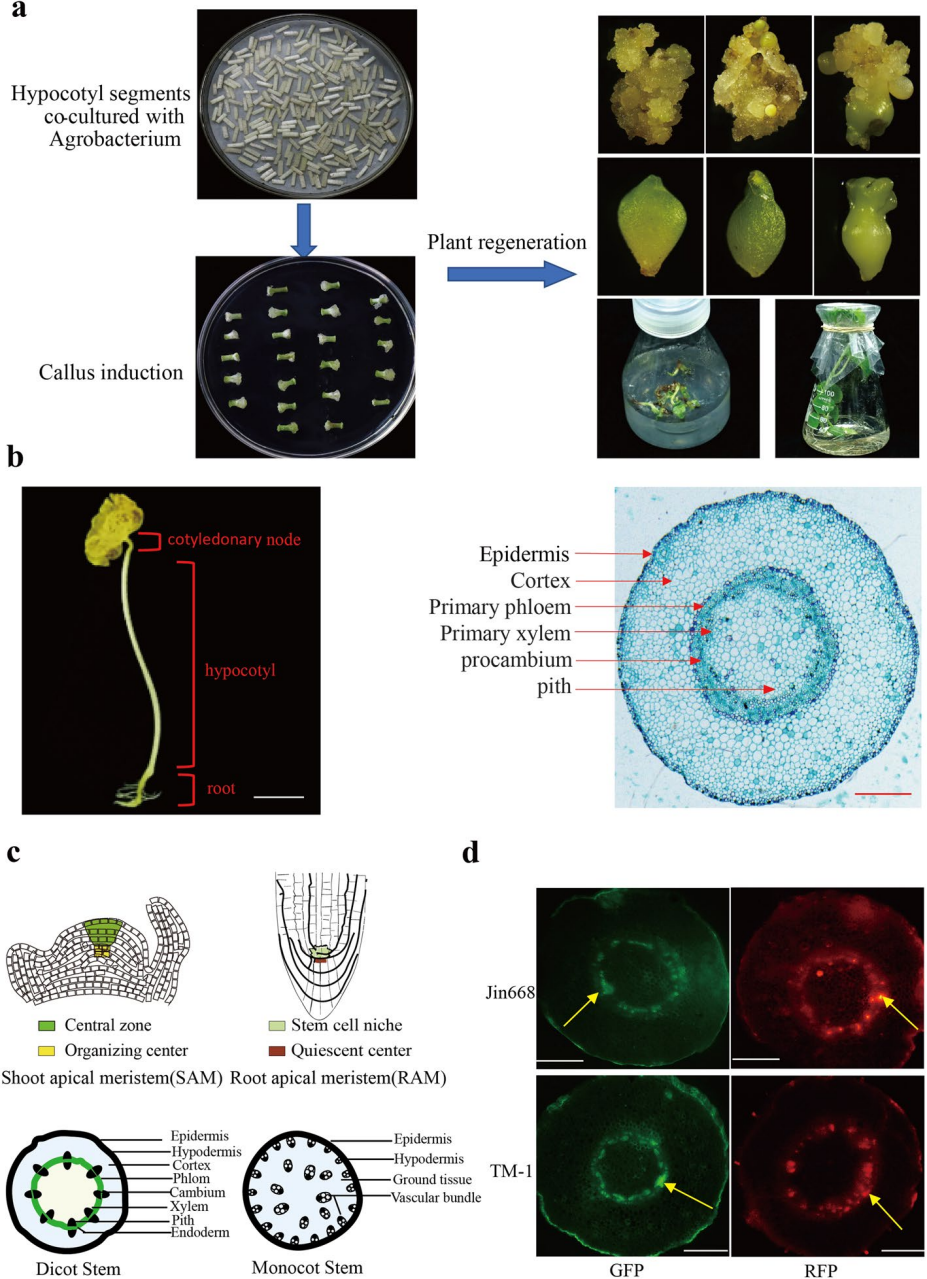
Primary vascular tissue of cotton hypocotyl is the primary target of *Agrobacterium* infection. **a** *Agrobacterium*-mediated genetic transformation and plant regeneration via somatic embryogenesis with cotton hypocotyls as explants, including callus induction, embryogenic callus induction, and plant regeneration via somatic embryogenesis. **b** The left picture shows etiolated cotton seedling under dark culture. Scale bar, 1 cm. The right picture shows the tissue types in the cross-section of cotton hypocotyl. Scale bar, 500 μm. **c** The comparison of three major types of stem cells in plants, including SAM, RAM, and cambium in vascular bundles. The vascular bundle of dicotyledons is cyclic annular and consists of xylem, phloem, and cambium. The vascular bundles of monocotyledons are scattered without cambium. **d** Specific expression of green fluorescent protein and far-red fluorescent protein driven by CaMV 35S promoter in primary vascular tissue (indicated by yellow arrow) after Jin668 and TM-1 hypocotyls infected with *Agrobacterium* for 48 h. Scale bar, 500 μm

Hypocotyl is the main explant material used for *Agrobacterium*-mediated genetic transformation of cotton and has no branch structure (Fig. 1a, b). Previously, we found that *Agrobacterium* mainly expresses the transgene in the primary vascular tissue of cotton hypocotyls, which then produces stably transformed callus and regenerated plants via somatic embryogenesis [10]. The cambium tissue of hypocotyl vascular bundles is arranged in rings, typical of dicotyledonous plants (Fig. 1c), and as a meristem is capable of continuous cell division [50]. After co-culture with *Agrobacterium* for 48 h under 19℃, the GFP (green-fluorescent protein) and RFP (red-fluorescent protein) under the transcriptional control of CaMV-35S promoter expression was detectable in the primary vascular tissue of the hypocotyls from both Jin668 and TM-1 (Fig. 1d). Transgene expression was mainly observed in the same tissues from both genotypes, suggesting that the primary vascular tissue of cotton hypocotyl is most competent to express transgenes, either because of its higher metabolic/transcriptional activity or because the cells are more cytoplasmically dense, compared to cortical parenchyma.

**Fig. 2:**
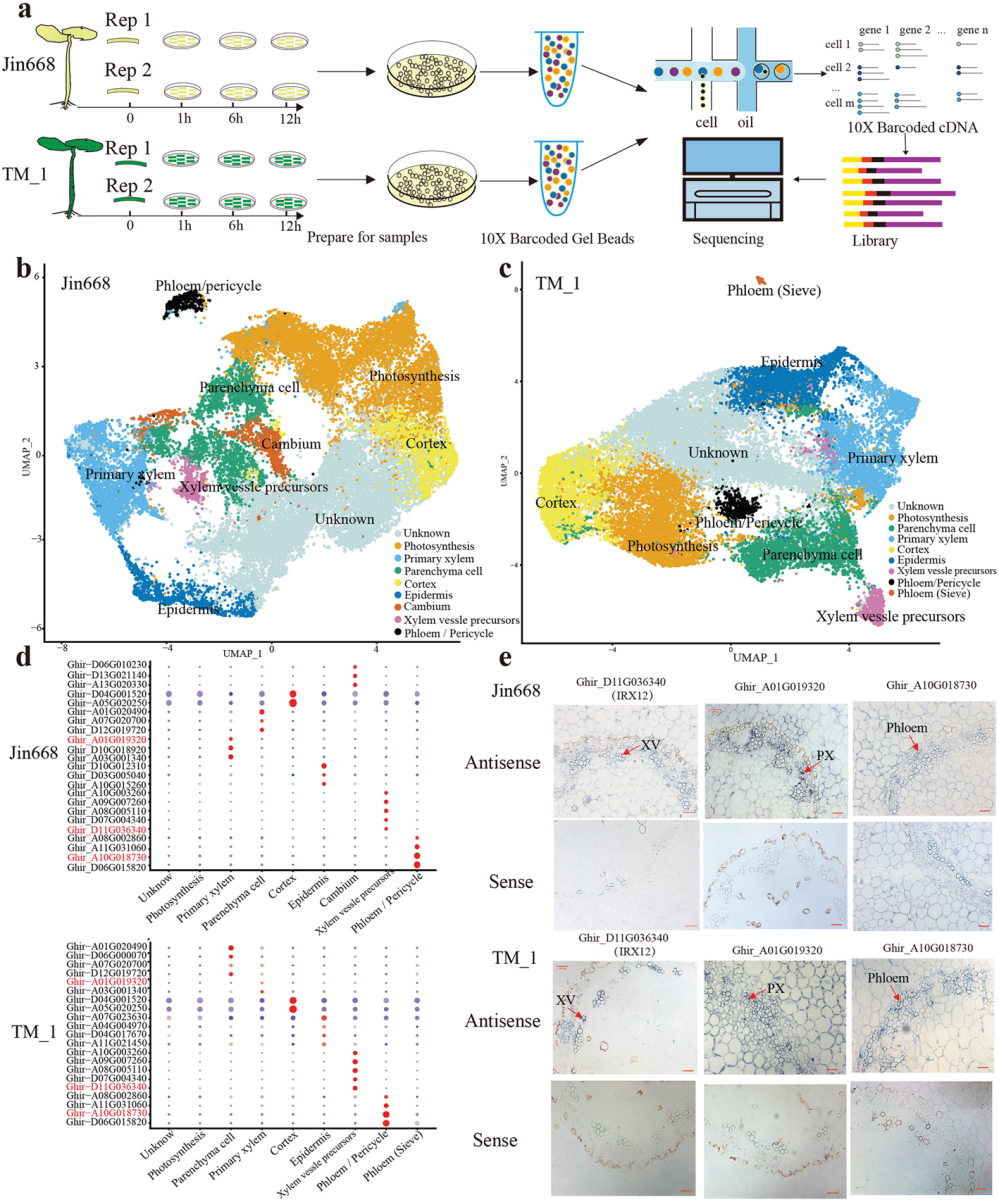
Single-cell RNA-seq and cluster annotation of cotton hypocotyl. **a** Overview of cotton hypocotyl scRNA-seq workflow. Protoplasts were isolated from hypocotyl sections of Jin668 and TM-1 with two independent biological repeats, respectively. 10 × Genomics platform was used for high-throughput sequencing. **b, c** UMAP visualization shows these cotton hypocotyl cells were grouped in to 9 clusters both in Jin668 and TM-1. Each dot indicates a single cell. Different colors indicate cell clusters. **d** Marker genes of each cell cluster in Jin668 and TM-1. **e** RNA in situ hybridization of 3 selected genes, including *Ghir_A01G019320* in primary xylem (PX), *Ghir_D11G036340* in xylem vessel precursors (XV), and *Ghir_A10G018730* in phloem (indicated by red arrow). Scale bar, 50 μm

scRNA-seq is an effective approach to gain insight into transcriptional activity in a spatial context and provides an approach to explore the process of cell fate transformation during somatic embryogenesis. We therefore used scRNA-seq to create a landscape of major hypocotyl cell types before and during callus induction. Single cells from cotton hypocotyl were isolated as protoplasts from four time points (0, 1, 6, and 12 h of tissue culture), with two independent replicates per time point (Fig. 2a). Microscopical analysis showed that most protoplasts were intact and active (Additional file 1: Fig. S1a). About 100,000 protoplasts per sample were then subjected to droplet-based scRNA-seq using the 10 × Genomics scRNA-seq platform. Following quality control at both cell and gene level, 30,357 cells for Jin668 (5836 cells at 0 HACI, 7364 cells at 1 HACI, 8575 cells at 6 HACI, and 8582 cells at 12 HACI) and 29,234 cells for TM-1 (7021 cells at 0 HACI, 7636 cells at 1 HACI, 8266 cells at 6 HACI, and 6311 cells at 12 HACI) were recovered and with high reproducibility between the biological replicates (Additional file 1: Fig. S1b; Additional file 2: Table S1). For subsequent cell clustering and annotation, two independent replicates were merged for each sample and analyzed independently. Unsupervised analyses with t-distributed stochastic neighbor embedding (*t*-SNE) or uniform manifold approximation and projection (UMAP) recovered distinct cell clusters of the hypocotyl in both TM-1 and Jin668 (Fig. 2b, c and Additional file 1: Fig. S2a,b). Visual inspection of the data for each time point by using *t*-SNE and UMPA suggested that there were some overlapping distributions of cells and a similar proportion of cell identities (Additional file 1: Fig. S3a). In addition, distribution of cell numbers and mRNA profiles in Jin668 and TM-1 was highly similar (Additional file 1: Fig. S3b; Additional file 3: Table S2).

### Seven major cell types identified in hypocotyl through marker gene expression

To assign cell identity to the clusters, marker genes were collected through Plant Single Cell Transcriptome Hub (PsctH; http://jinlab.hzau.edu.cn/PsctH/) [51] (Additional file 4: Table S3). The top 50 marker genes for each cell cluster for Jin668 (named Jin0 to Jin8) and TM-1 (named TM0 to TM8) were used to match the cell type. Orthologs of the marker genes were identified and used to annotate the clusters (Fig. 2d and Additional file 4: Table S3). This strategy allowed us to roughly identify major cell types in the hypocotyl of Jin668 and TM-1, i.e., the epidermis, cortex, xylem vessels, primary xylem, primary phloem, cambium, and parenchyma. Specifically, several marker genes related to phloem identity were highly enriched in Jin7 and TM7 and include *ATSUC2* [52] and the *PHLOEM PROTEIN 2* family *ATPP2-A9* (*Ghir_D06G015820*). As *ATPP2-A1* (*Ghir_D06G022070*) [53] were enriched in TM7, this cluster was identified as phloem/pericycle (Additional file 1: Fig. S4a). Compared with Jin668, TM-1 has more mature phloem sieve tube cells. Several homologous genes of *SIEVE-ELEMENT-OCCLUSION-RELATED 1* (*SEOR1*) (*Ghir_D03G011280*, *Ghir_D10G009100*), which are necessary for the formation of phloem during *Arabidopsis* somatic embryogenesis [54], were particularly enriched in cluster TM8 (Additional file 1: Fig. S4a). Accordingly, the cells in clusters TM8 were identified as sieve elements. Likewise, *ATCTL2* (*Ghir_A09G007260*), related to xylem development [55], and the xylem biosynthesis and deposition genes *IRX1* (*Ghir_A10G003260*), *IXR1* (*Ghir_A08G005110*), *IRX12* (*Ghir_D11G036340*), and *IRX3* (*Ghir_D07G004340*) [56] were highly enriched in clusters Jin6 and TM6, suggesting that they are from xylem cells (Additional file 1: Fig. S4a). *TIP2;2*, associated with water transport, is highly expressed in the Jin2 cluster and is also found in TM2. *GAMMA-TIP*, involved in gibberellic acid mediated signaling pathway, is specifically expressed in the vascular tissues of *Arabidopsis* and mainly distributed in Jin2 and TM2 clusters. Therefore, cells in these two clusters were identified as primary xylem. Meanwhile, genes involved in cell differentiation and cell division, such as *FLA2* (*Ghir_A07G020700*), were found in Jin3 and TM3 clusters, suggesting that Jin3 and TM3 clusters may be xylem parenchyma cells. Further analysis of the enriched genes related to cell cycling and to the development of primary xylem [57, 58], such as *PRXR1* (*Ghir_D05G017420*) and *AP40* (*Ghir_A10G004910*), were strongly expressed in Jin8 clusters (Additional file 4: Table S3). *TIP4;1* (*Ghir_D06G010230*) and *SBT1.8* (*Ghir_D13G021140*), were predicted to be involved in division and epidermal cell development [59]. These data indicated that cell cluster Jin8 comprises dividing cells to establish the cambium and adjacent differentiating cells. In addition, the meristem regulation-related gene *SHD* (*Ghir_D06G000070*) was highly enriched in TM3, suggesting some TM3 cells are associated with the cambium.

In order to identify epidermal cell populations, *AT2G38540* (*Ghir_A10G015260*) (specifically expressed in L1 epidermal layer of *Arabidopsis* [60]), was chosen as the relevant marker gene and was identified in Jin5 clusters. *CYP82C4* (*Ghir_A05G020250*) (expressed in root cortex) [61] was highly enriched in clusters Jin4 and TM4, identifying these two clusters as cortex cells. Interestingly, many genes involved in response to stress were also predominantly expressed in clusters Jin4 and TM4, including *ATPA2* (*Ghir_A08G024060*), *MSS1* (*Ghir_A09G022880*) (response to water deprivation), and *NHL3* (*Ghir_A09G014390*) (response to bacteria) (Additional file 4: Table S3). For cell clusters Jin1 and TM1, the genes enriched in these two clusters were mainly related to photosynthesis, indicating that this cell type may be photosynthetic. Due to the lack of proven marker genes, we could not determine the cell types in clusters Jin0 and TM0. However, the transcriptome profiles analysis of these two clusters revealed a correlation with the stress response.

To confirm the accuracy of cell cluster classification, we selected genes both expressed in Jin668 and TM-1 as marker genes for RNA in situ hybridization. Among them, *Ghir_A01G019320* and *Ghir_A10G018730* were newly identified marker genes, which was expressed in the primary xylem and phloem of cotton hypocotyls, respectively (Fig. 2e). Besides, some genes enriched in Jin668 cell clusters are also expressed in the same site of TM-1. These data indicate that the cell types we identified are correct and Jin668 and TM-1 have the same cell type **(**Additional file 1: Fig. S4d).

These results reveal the high degree of cell heterogeneity in hypocotyl. Overall, multiple tissues, including cortex, epidermis, xylem, and phloem contributed to the major clusters containing cells ranging in number from 500 to 4000. The scRNA-seq dataset from Jin668 and TM-1 hypocotyl allowed us to explore the mechanism of plant cell proliferation at single-cell resolution and the resultant dataset can be mined interactively on the Web using the Cell Landscape in Cotton (CLC), which is publicly available at http://jinlab.hzau.edu.cn/CLC/ or http://jinlab.hzau.edu.cn:8000/CLC/.

### Primary vascular cells of cotton hypocotyl are the main cell types for initiating cell reprogramming during somatic embryogenesis

**Fig. 3:**
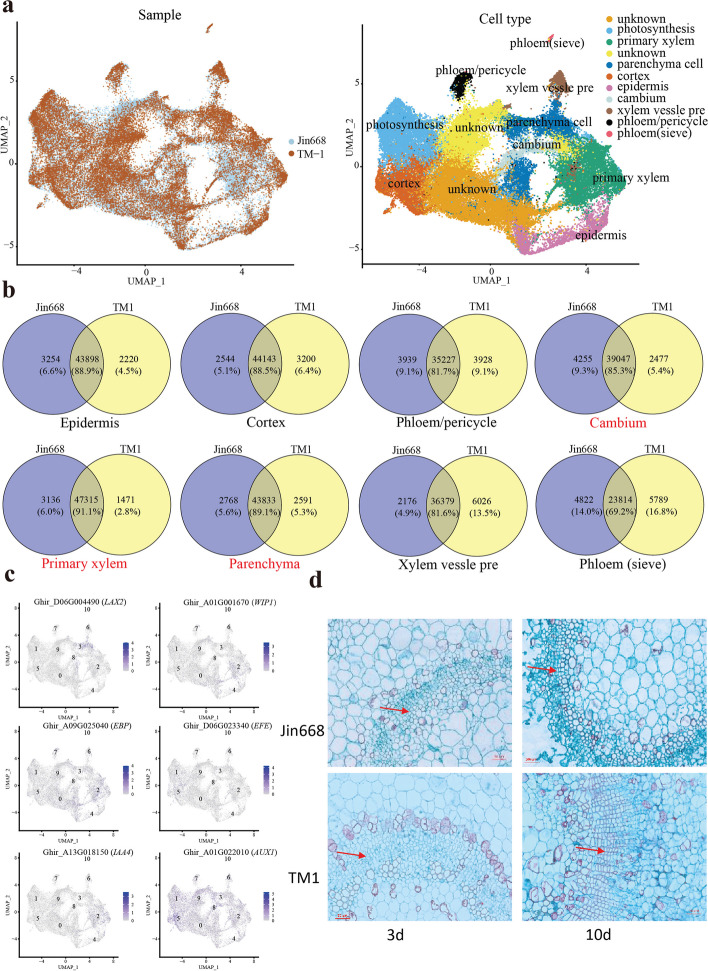
Highly conserved cell-type clusters and heterogeneity genes between Jin668 and TM-1. **a** UMAP visualization of Jin668 and TM-1 clusters after alignment. The left is sample (Jin668 or TM-1), the right is cell type. **b** Venn diagram showing the number of shared and cultivar-specific expressed genes for each cell-type cluster of Jin668 and TM-1. **c** UMAP visualization of expression patterns of the genes related to SE, including auxin and wound response. The colors represent expression levels of these genes in individual cells. **d** Paraffin section showed vascular tissue proliferation of hypocotyl after induction. The red arrows represent the proliferation site

Although the two upland cotton genotypes TM-1 and Jin668 have similar agronomic traits, they exhibit distinct responses during *Agrobacterium*-mediated transformation and the subsequent tissue culture process. In the process of *Agrobacterium*-mediated genetic transformation, hypocotyls from Jin668 can undergo dedifferentiation and re-differentiation to form embryogenic callus and finally regenerate plants via somatic embryogenesis (Fig. 1a), while TM-1 can only produce non-embryogenic callus that cannot undergo somatic embryogenesis and plant regeneration. We first wanted to investigate how different cell types respond to external stimuli during callus induction. To this end, all the single-cell data from TM-1 and Jin668 were merged (termed “Cotton”) and subsequently clustered using *t*-SNE and UMAP. Then, these cell-type clusters were aligned (Fig. 3a). To further characterize the clusters, we identified conserved and differentially expressed genes (DEGs) for the homologous cell types. Venn diagram analysis showed more than 80% of co-expressed genes were shared between TM-1 and Jin668 in all major cell-type clusters, including epidermis, cortex, and primary vascular tissue (Fig. 3b), except for phloem sieve elements, which showed the highest proportion of genes specific to each genotype (about 15%) (Fig. 3b).

To identify cell types involved in somatic embryogenesis, the expression pattern of genes involved in auxin synthesis and response, wound induction, cytokinin, and epigenetic regulation were checked in the merged clusters (Fig. 3c). For these genes, they were found to be highly expressed in primary vascular tissue cells (Cotton2, Cotton3, and Cotton8, Fig. 3c and Additional file 1: Fig. S4b). For example, a member of the *LAX* auxin transporter family, *LAX2* (*Ghir_D06G004490*), a gene required for the regulation of vascular patterning, is mainly expressed in the identified cluster Cotton3 (parenchyma cells). A wound response family protein *WIP1* (*Ghir_A01G001670*), *IAA* transcriptional regulatory family protein *IAA4* (*Ghir_A13G018150*), and an ethylene response element binding protein *EBP* (*Ghir_A09G025040*) were highly expressed in Cotton2 (primary xylem), but not in mature xylem (Cotton6). However, another auxin response family protein gene, *EFE* (*Ghir_D06G023340*), was enriched in the Cotton5 cluster (cortex) and the auxin transporter gene *AUX1* (*Ghir_A01G022010*) was active in Cotton8 (cambium). Histological sections showed that the hypocotyl cambium began to proliferate after three days of induction on the medium (Fig. 3d). Therefore, we proposed that those xylem parenchyma cells are more sensitive to hormone responses as induced embryogenic cells (IEC). The cell type with stronger meristematic activity (cambium in Jin668 and some cells of parenchyma in TM-1) is defined as pre-embryogenic cells (PEC). These results suggest that primary vascular tissue, especially the cambium and vascular parenchyma, is the major region that responds to hormone induction during callus induction.

### Primary vascular cells of Jin668 hypocotyl express specific genes and show a different auxin response gene expression pattern compared to TM-1

**Fig. 4:**
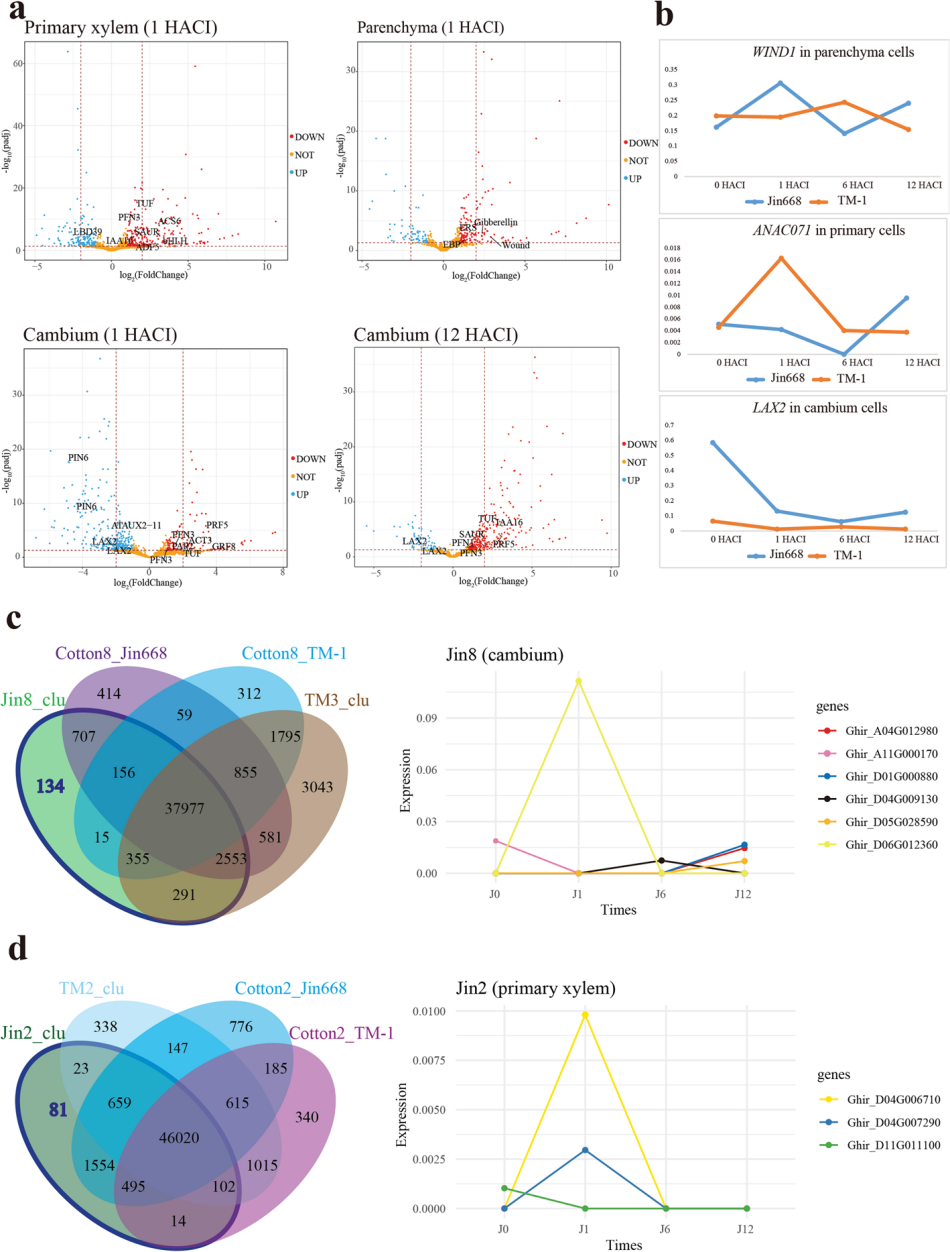
Genes specifically expressed in primary vascular tissue cells of Jin668 and TM-1. **a** Differential expression gene (DEGs) analysis between Jin668 and TM-1 after induction at the same time. Colored by fold-change direction (padj ≤ 0.05 & log2FoldChange ≥ 1). **b** Gene expression trend analysis showed that the auxin influx gene *LAX2*, wound response gene *WIND1*, and *ANAC071* had a reverse expression trend in Jin668 and TM-1. **c, d** Venn diagram showing the genes specifically expressed in the cambium (**c**) and primary xylem (**d**) of Jin668. The expression trend of auxin transport-related genes after induction at different times in cambium (**c**) and primary xylem (**d**)

The similarity in cell cluster separation between TM-1 and Jin668 promoted us to investigate the conservation and heterogeneity of their single-cell transcriptomes. To this end, the gene expression profile was investigated and compared between Jin668 and TM-1 in the same cell type, at the same induction time, to further understand cell fate transitions into embryonic development. The results reveal multiple DEGs in different cells. The DEGs in epidermis (556 genes), cortex (245 genes), and phloem (140 genes) were mostly related to their functions in the respective cell types. In primary xylem, about 1% of DEGs (968), 1% in xylem parenchyma (415), and 2% in the cambium (1040) were related to plant regeneration (Additional file 5: Table S4). Among them, many genes are related to hormone responses, including *ACT3*, *EMB2448*, *ERS*, and *EBP* (Fig. 4a). Therefore, we considered that these cell types are the major cell types that undergo fate changes during the early stages of callus induction prior to somatic embryogenesis. When the traditional bulk-RNA-seq-method is used to process these scRNA-seq data and compare gene expression between TM and Jin668 at the same time point, we identified a total 3749 DEGs (694 DEGs at 0HACI, 1772 at 1HACI, 12 at 6HACI, and 1271 at 12HACI). Compared to traditional bulk-RNA-seq-method, scRNA-seq can identify DEGs more precisely in time and space during development (Additional file 1: Fig. S4e).

In order to understand better the spatio-temporal expression patterns for genes within the xylem and parenchyma cell types of Jin668 and TM-1, the gene expression profiles and expression trends at four time points (0, 1, 6, and 12 HACI) were investigated and compared (Additional file 1: Fig. S5a). Based on the fact that Jin668 can achieve regeneration but TM-1 cannot, we focused on genes with opposite trends of expression in the corresponding genotype cell clusters. Embryogenic cell marker gene *SERK1* (*Ghir_D13G005690*) was detected in the three cell types, and increased in the primary xylem of Jin668, but decreased in the equivalent cell types of TM-1 (Additional file 1: Fig. S4c). Wound induction-related genes *WIND1*(*Ghir_A06G003880*) and *ANAC071* (*Ghir_A12G022250*) were expressed both in Jin668 and TM-1. Among them, *WIND1* was mainly expressed in the parenchyma and cambium cells and was up-regulated in Jin668 and down-regulated in TM-1. While *ANAC071* has different expression trends in primary cells of Jin668 and TM-1. Auxin response-related genes *AHL15*, *LBD16*, *LBD18*, and *LBD19*, auxin-responsive factors *ARF19* and *E2Fa*, and the auxin transport-related gene *TET3* were expressed in primary xylem and parenchyma clusters. *TET3* was mainly expressed in parenchyma cells, with completely opposite trends in Jin668 and TM-1. *AUX1/LAX*-dependent auxin influx is necessary for the formation of cellular patterning from early embryogenesis onward [62]. We found that *LAX2* (*Ghir_D01G017380*) is only expressed in cambium cells and exhibits a distinctly different expression pattern in Jin668 and TM1, with up-regulation in Jin668 and down-regulation in TM-1 after 6 HACI (Fig. 4b).

To identify genes specifically expressed in Jin668, we compared the data for Jin668 and TM-1 to obtain genes that are specifically expressed in primary xylem, cambium, and parenchyma of Jin668 (Fig. 4c, d and Additional file 1: Fig. S5b) and then performed functional annotation of these genes. In the primary xylem, 81 genes were identified as specifically expressed in Jin668, including the cytokinin metabolic-related gene *CKX3* (*Ghir_A07G001250*), abscisic acid biosynthetic gene *ICE1* (*Ghir_A03G006670*), auxin synthesis-related gene *PLC2* (*Ghir_D04G007290*), and auxin polar transport gene *PIS1* (*Ghir_D11G011100*). In the cambium region of Jin668 we detected auxin-related genes *MYB44* (*Ghir_D01G000880*), *MYB73* (*Ghir_A04G012980*), *CSEF* (cotton somatic embryogenesis factor; *Ghir_A11G000170*), *PIS1* (*Ghir_D04G009130*), ethylene response-related genes *RAP2.11* (*Ghir_D13G022910*), *PDR12* (*Ghir_D05G012460*), *FER* (*Ghir_D07G019570*), wounding response gene *Ghir_D12G014280*, *Ghir_A06G021760*, and gibberellic acid homeostasis gene *Ghir_D05G004470*. *PLT3* (*Ghir_D10G010630*), which is essential for maintaining high levels of *PIN1* expression at the meristem and in the central region of the SAM, was primarily expressed in parenchyma cells. Interestingly, auxin transport-related genes were down-regulated in all three cell types of Jin668 (Fig. 4c, d). Given the auxin influx gene, *AUX/LAX* is enriched and up-regulated in Jin668 polar transport and distribution of auxin may be important in cells undergoing re-differentiation, and these Jin668 cells may be more likely to produce callus and form somatic embryos under inductive conditions.

### Reconstruction of the continuous differentiation trajectory of hypocotyl vascular cells during callus induction

**Fig. 5:**
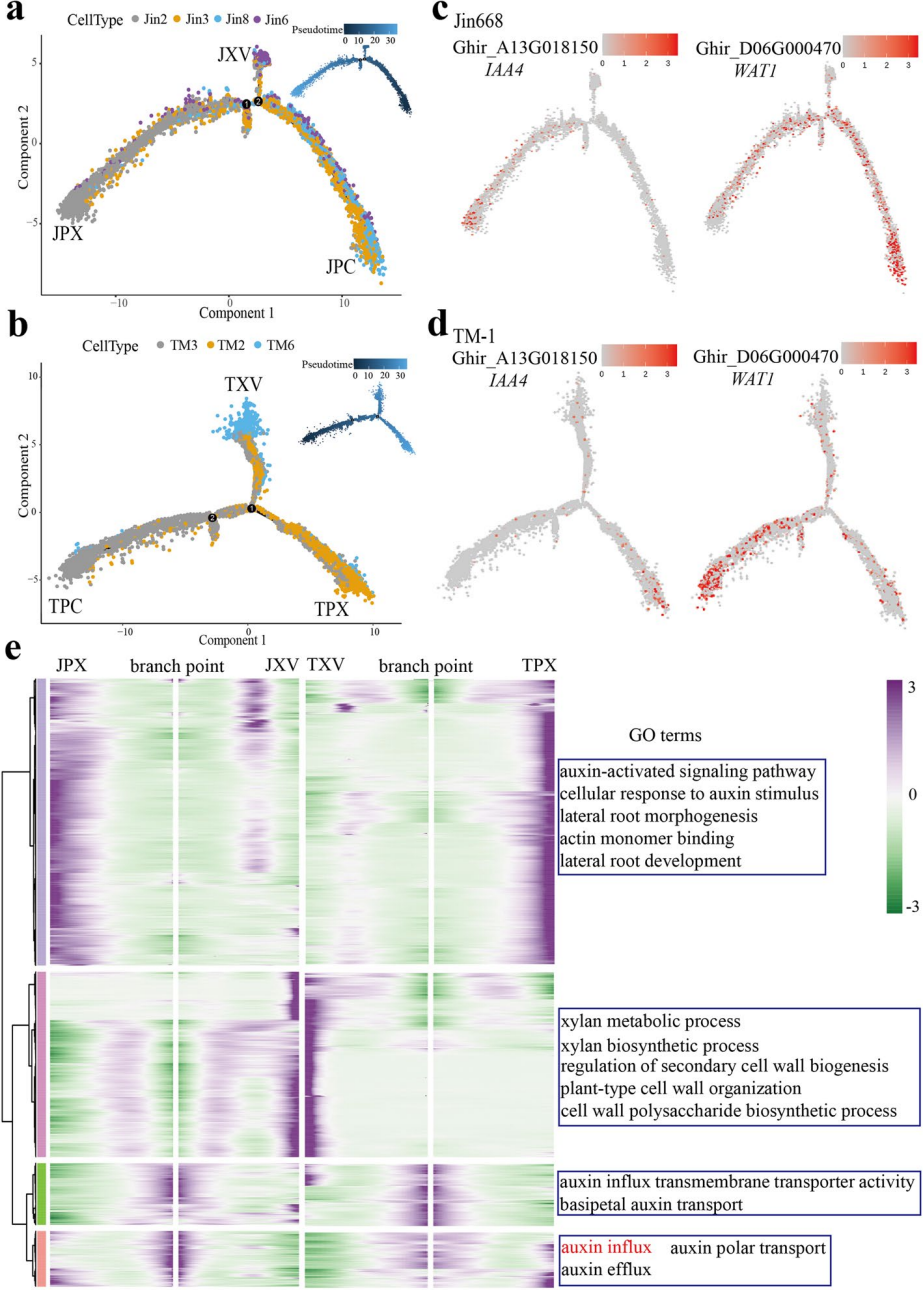
Pseudotime trajectory of primary vascular cells. **a, b** Pseudotime analysis of primary xylem, xylem vessel precursors, parenchyma, and cambium cells of Jin668 and TM-1. Each dot indicates a single cell, and the color of the upper right corner represents the starting point and end point of differentiation. **c, d** Expression patterns of representative auxin-related genes (*WAT1* and *IAA4*) are shown over the course of pseudo-time. Color bar indicates the relative expression level. **e** Heatmap showing the expression of the branch-dependent genes over pseudo time. GO terms of SE-related are shown in the table on the right. The middle of the heatmap is the beginning of pseudo time. Both sides of the heatmap are the end of pseudo time. Color bar indicates the relative expression level. J(T)PX, primary xylem of Jin668 (TM-1); J(T)XV, xylem vessel precursors of Jin668 (TM-1); J(T)PC, parenchyma and cambium region of Jin668 (TM-1)

The scRNA-seq data enable us to explore the developmental trajectory of cells during the time-course of callus induction. As described above, cell fate transition-related genes are enriched in primary vascular tissue (Fig. 3c and Additional file 1: Fig. S4b). To provide a reconstructed trajectory of hypocotyl vascular cells during callus induction, pseudo time analysis was applied to the clusters representing these cell types using Monocle2. By using the data from parenchyma cells, cambium, and primary xylem, we produced a representation of the distinct final states of the clusters, arranged at different developmental branch sites. In Jin668, the differentiation started in cambium and parenchyma cells, with primary xylem and xylem vessel precursors grouped into different branches. These states are named as JPC (Jin668 parenchyma cells/cambium), JPX (Jin668 primary xylem), and JXV (Jin668 xylem vessel precursors), respectively (Fig. 5a). The trajectory differentiation of TM-1 is similar to Jin668, the starting branch being TPC (TM-1 parenchyma cells), and the final branch being TPX (TM-1 primary xylem) and TXV (TM-1 xylem vessel precursors) (Fig. 5b).

The spatial distribution of auxin plays an important role in the maintenance of the cambium [63]. Gene Ontology (GO) enrichment analysis found that the genes mainly enriched at the starting point of the pseudo-time trajectory included “auxin efflux”, “auxin polar transport”, and “auxin transmembrane transporter activity” (Fig. 5e). The auxin transport-related genes *WAT1* (*Ghir_D06G000470*) and *IAA4* (*Ghir_A13G018150*) were enriched at the beginning of the branch point in both Jin668 and TM-1 cell clusters, supporting the view that auxin transport is involved in regulating cambium cell activity (Fig. 5c, d). We identified significantly higher numbers of DEGs in Jin8 and TM3. Notably, the auxin influx carrier genes *LAX1* (*Ghir_D04G013960*, *Ghir_A09G017300*) and *LAX2* (*Ghir_ D06G004490*) were only expressed in the start branch of Jin668, suggesting that the cambium cells of Jin668 and TM-1 may differ in auxin distribution patterns during callus induction.

During somatic embryogenesis, auxin plays an important role in the establishment of polarity in somatic embryos and auxin transport-related genes affect the acquisition of embryogenic abilities of somatic cells. Auxin polar transporter gene *PIN1* (*Ghir_D12G028380*, *Ghir_A09G018310*) is mainly distributed in the initial branches and primary xylem on the trajectory (Additional file 1: Fig. S5c). This means that there may be dynamic changes in auxin flow between these cells and the distribution of auxin may affect the fate of cells. In order to explore the differentiation trajectory of different tissues, we further investigated differences in gene expression patterns between XV (xylem vessel precursors) and PX (primary xylem) branches. The results showed that in the XV branch, both TM-1 and Jin668 were enriched in “xylan metabolic process”, “regulation of secondary cell wall biogenesis”, and “plant-type cell wall organization” (Fig. 5e), indicating that these cell types are undergoing cell wall thickening and lignification. Interestingly, transcripts preferentially expressed in JPX and TPX are enriched for “lateral root development”, “lateral root morphogenesis”, “response to growth hormone”, “auxin-activated signaling pathway”, and “cellular response to auxin stimulus”. Then we investigated in detail the cell development trajectory of JPX with induction time at 0, 1, 6, and 12 HACI (Additional file 1: Fig. S6a). In the final cell state, there are wound-induced cell responses, including the accumulation of ROS, ethylene, and jasmonic acid (JA) related genes (Additional file 1: Fig. S6b). Therefore, we speculate that in the process of somatic embryogenesis, cells that are less differentiated, such as cambial cells, are more likely to undergo dedifferentiation to form callus and obtain embryogenic capacity.

We also analyzed the DEGs that might affect dedifferentiation in the two genotypes. Auxin-related genes *PIN3* (*Ghir_D01G015820*), *AFB2* (*Ghir_D07G024880*), *ATHB2* (*Ghir_A12G022820* and *Ghir_D11G002820*), and *CSEF* (*Ghir_A11G000170*) were especially strongly expressed in JPX of Jin668 compared to TM-1, implying that these genes may be involved in xylem cell dedifferentiation (Fig. 5e and Additional file 4: Table S3).

In conclusion, we propose that callus originates principally from primary xylem cells, and cambium cells may play an important role in maintaining auxin balance. There is likely intensive cell communication between cells of the primary xylem and cambium, which affects the formation of callus and subsequent regeneration of plants.

### RNA velocity field describes fate decisions of major cells in cotton hypocotyl transition to primary xylem cells

**Fig. 6:**
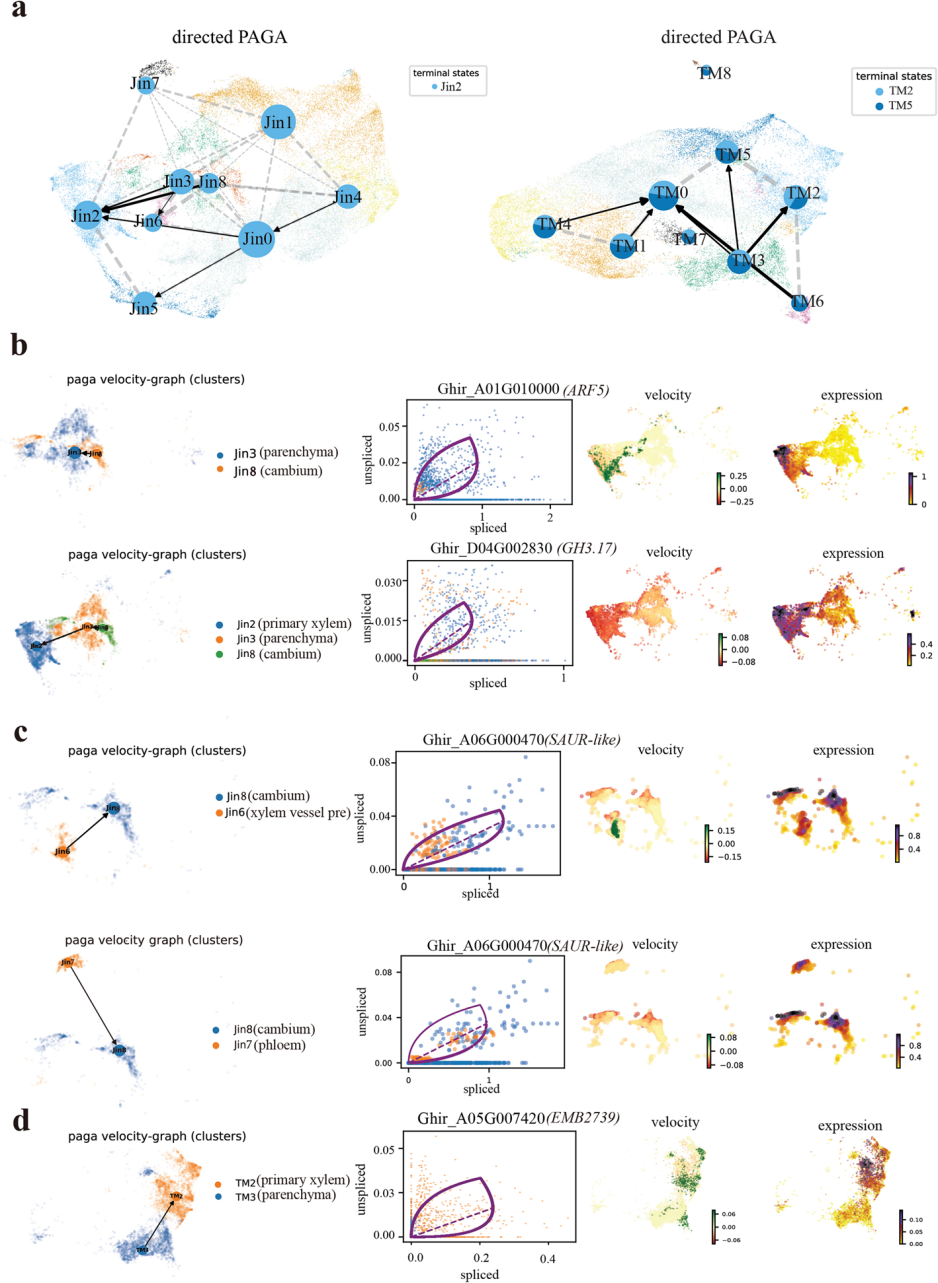
RNA velocity field describes fate decisions of primary vascular cells in the hypocotyls of Jin668 and TM-1. **a** Aggregated the fate maps of cell clusters in Jin668 and TM-1 into primary vascular cells using directed edges. The pie charts to show cell fates averaged per cluster. Edges between clusters are given by transcriptomic similarity between the clusters. **b–d** The fate maps of directional aggregation and gene expression trend of local cell clusters. Fate maps, phase portraits, unspliced residuals, and smoothed gene expression trends are shown from left to right for these driver regulated genes

RNA velocity methods [64], which leverage the intrinsic RNA splicing process, were used to quantify cellular transitions during tissue culture and reveal regulatory genes in Jin668 and TM-1. In Jin668, RNA velocity showed a strong directional flow toward primary xylem cells, while in TM-1, velocity was toward primary xylem and epidermis cells (Fig. 6a). As expected, pre-cambium cells trend toward primary xylem cells, while across parenchyma cells (Fig. 6b). Phase portraits showed specific induction and repression of gene expression along the manifold. For example, the AUX-IAA gene *ARF5* (*Ghir_A01G010000*) and *GH3.17* (*Ghir_D04G002830*) (Fig. 6b and Additional file 1: Fig. S7a) showed positive velocity from cambium cells to primary xylem, suggesting that it may play an important role in guiding xylem cells to form callus under hormone induction. Interestingly, mature cells types such as xylem and phloem were also found to exhibit a tendency toward pre-cambial identity (Fig. 6b), indicative of the dedifferentiation process. In addition, we found that *SAUR51* (*SAUR*-like auxin-responsive protein) exhibited the most obvious change during this process, also indicative of a role in xylem and phloem cell dedifferentiation (Fig. 6c). In contrast, in TM-1, parenchyma cells/cambium region had a velocity trending toward primary xylem. The embryo development-related gene *EMB2739* [65] (*Ghir_A05G007420*) and root development-related gene *Ghir_A10G019630* were expressed in final states, which means that primary xylem of TM-1 may eventually differentiate into root cells rather than callus or somatic embryo (Fig. 6d and Additional file 1: Fig. S7b). Collectively, RNA velocity and pseudo time analysis provided novel insight into the dynamic process of cell-type development and gene expression rewiring during the transition of cell states in cotton hypocotyl tissue culture.

### scWGCNA analysis provides new insights into cell–cell interactions to regulate vascular cell differentiation and plant regeneration

**Fig. 7:**
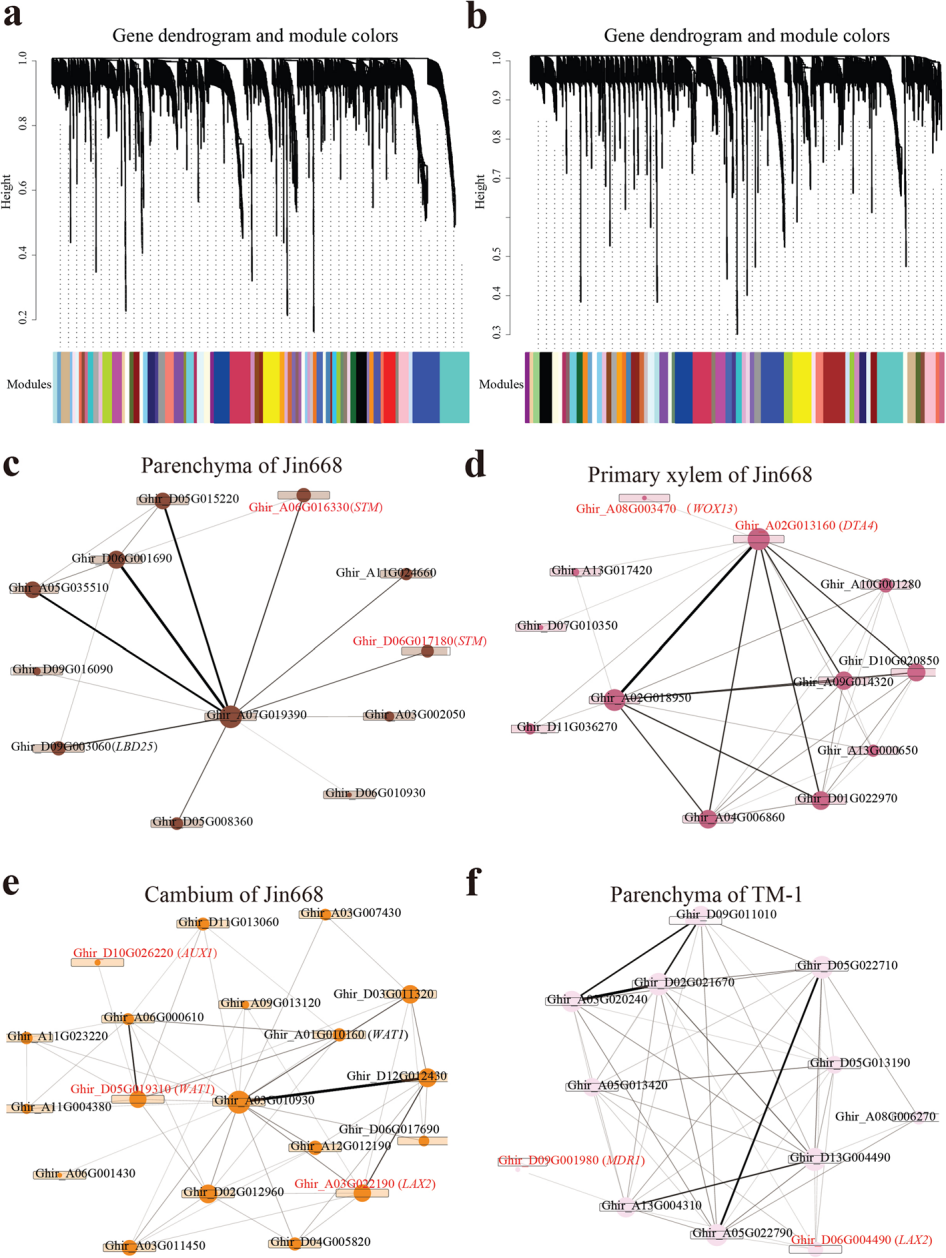
Network analysis SE-related genes of Jin668 and TM-1 hypocotyl primary vascular cells. **a, b** Weighted correlation network analysis of scRNA-seq data of Jin668 (**a**) and TM-1 (**b**) reveals multiple modules of co-expressed genes of various sizes. The color bar beneath the dendrogram represents the module assignment of each gene. **c–f** Module visualization of network connections and associated function in Jin668 and TM-1. The reported SE-related intramodular hub genes are indicated by a red dot

Pathways rather than the expression individual genes are the functional units of cells [66]. To systematically investigate the genetic program dynamics in cultured tissues of Jin668 and TM-1, weighted gene co-expression network analysis (WGCNA) was performed (Additional file 1: Fig. S8). WGCNA identified 69 gene modules for Jin668 and 60 for TM-1 (Fig. 7a, b), and each module contains a set of genes that tend to be co-expressed in particular cell types (Additional file 1: Fig. S9a, b). As described previously, we mainly focused on primary xylem, parenchyma, and cambium cells. The results showed that the *STM* genes (*Ghir_A06G016330* and *Ghir_D06G017180*) related to meristem maintenance and which are required for the establishment of embryonic polarity, are specifically expressed in the regulatory network of the Jin668 parenchyma module. In addition, *STM* (*Ghir_A06G016330* and *Ghir_D06G017180*) in the parenchyma of Jin668 module regulate somatic embryogenesis in combination with *Ghir_D06G001690* and auxin-related *LBD25* (*Ghir_D09G003060*) (Fig. 7c), which implies again that there are different auxin polarity establishment networks in Jin668 and TM-1. In the primary xylem of Jin668, *WOX13* (*Ghir_A08G003470*) and *DTA4* (*Ghir_A02G013160*) were co-expressed, which was not the case in TM-1 (Fig. 7d). Notably, the previously mentioned auxin transport-related genes *AUX1* (*Ghir_D10G026220*), *LAX2* (*Ghir_A03G022190*), and *WAT1* (*Ghir_A01G010160*, *Ghir_D05G019310*) were in the same regulatory network in the cambium cells of Jin668 (Fig. 7e). In the same cell type of TM-1, *LAX2* (*Ghir_D06G004490*) and the auxin polarity transport-related gene *MDR1* (*Ghir_D09G001980*) are in the same co-expression network (Fig. 7f). These data indicate that different genes participate in the regulation of auxin influx in Jin668 and TM-1, adding to the evidence that Jin668 and TM-1 cells have different auxin transport properties during tissue culture, associated with different cell fate trajectories.

### Functional analysis of three genes identified in scRNA-seq by CRISPR/Cas9 knock out and overexpression

**Fig. 8:**
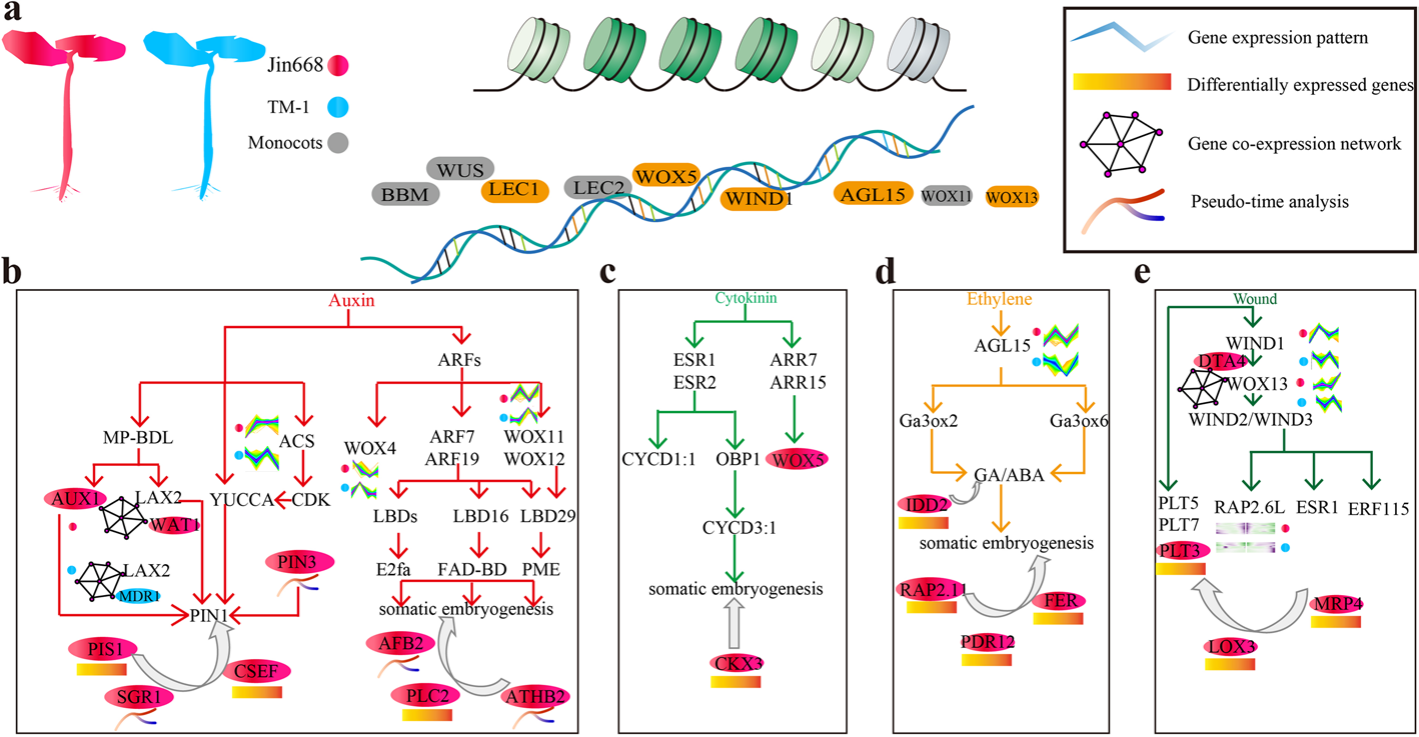
A proposed model of the molecular regulation of cotton plant regeneration via somatic embryogenesis. **a** Chromatin-modifying proteins repress or restrict expression of transcription factors (TFs) during SE. Red represents Jin668, blue represents TM-1, and gray represents monocotyledon. The symbols in the box represent the main differences between Jin668 and TM-1. It includes gene expression pattern, differential expressed genes, gene co-expression network, and pseudo-time analysis. **b–e** The main factors affecting SE and regulatory network of related genes. Including auxin (**b**), cytokinin (**c**), ethylene pathways (**d**), and wound induction process (**e**). The reported and newly identified regulatory genes under the single-cell resolution in this study based on gene differential expression, cell trajectory inference, RNA velocity fate determination, and co-expression gene interaction are labeled in the corresponding module. The red oval represents the gene specifically expressed in Jin668, and the gray arrow represents the predicted SE-related genes. Overall, Jin668 and TM-1 exhibited distinct regulation model including 20 core genes (such as *LAX2, WOX4, AGL15, WIND1*) during the plant regeneration process via somatic embryogenesis

We constructed a comprehensive regulatory network of plant regeneration-related genes combined with the single-cell landscape of hypocotyl cells for Jin668 and TM-1 (Fig. 8). This integrates data from multiple analysis, such as differential gene expression (Figs. 3 and 4), vascular cell trajectory inference (Fig. 5), RNA velocity fate determination (Fig. 6), and gene co-expression interactions (Fig. 7). Firstly, the chromatin opening state of totipotent transcription factor (TF) gene sites (such as *LEC1*, *BBM*, *LEC2*, *WUS*) is a prerequisite for somatic reprogramming. These genes were rapidly induced by hormone (Fig. 8a). Subsequently, TFs related to cell totipotency can induce the biosynthesis of auxin, cytokinin, ethylene, and wound response, thus strengthening the transformation of cell fate. Our results showed that the ethylene synthesis-related gene *ACS* (*Ghir_D11G009870*) of Jin668 was up-regulated in the cambium cells after induction, while in TM-1 was down-regulated. Therefore, we speculated that the up-regulated expression of ethylene synthesis-related gene *ACS* in cotton hypocotyls under a certain concentration of 2,4-D induction might promote the expression of auxin synthesis-related gene *YUCCA*, thus causing the increase of endogenous auxin levels in cambium cells (Fig. 8b). In addition, compared with TM-1, the auxin influx protein in Jin668 was up-regulated in primary xylem and cambium cells, and the auxin polar transport-related gene *PIN3* was specifically expressed in the primary xylem of Jin668. The auxin influx genes *AUX1*, *LAX2*, and efflux protein *PIN3* interact to promote the polar transport of auxin between primary xylem, parenchyma cells, and cambium cells. At the same time, *SGR1* and *CSEF*, expressed in cambium cells of Jin668, interact to maintain and regulate the fate of stem cells. The *PIS1* gene specifically expressed in the primary xylem was down-regulated to form a local maximum of auxin in the xylem, thus inducing the primary xylem cells to produce callus. Another pathway of auxin is to activate the expression of *LBD* family transcription factors *LBD16*, *LBD17*, *LBD18*, and *LBD29* through ARF transcription factors. These transcription factors in turn induce the expression of *E2Fa*, a transcription factor that plays a central role in cell cycle re-entry. We found that *AFB2* (*Ghir_D07G024880*), *ATHB2* (*Ghir_A12G022820* and *Ghir_D11G002820*), and *PLC2* were specifically expressed in Jin668, making Jin668 more sensitive to auxin (Fig. 8b).

In the process of plant somatic embryogenesis, auxin does not work independently in most cases, but regulates plant somatic embryogenesis together with cytokinin. Cytokinin upregulates the expression of *AP2/ERF* transcription factor *ESR1* and its functional homolog *ESR2* and mediates cell cycle reactivation. *ESR2* induced *CYCD1;1* and the *DOF* binding transcription factor *OBP1*. *OBP1* promotes cell cycle progression by inducing the expression of *CYCD3;1*, thereby affecting somatic embryogenesis. In addition, cytokinin activates type-B ARR transcription factor, and type-B ARRs and *WOX5* interact to promote plant regeneration. We found that *WOX5* (*Ghir_D10G026130*) were just expressed in Jin668 through Venn diagram analysis, which is conducive to promote the expression of cell cycle-related genes, thus enhances the division and proliferation of callus (Fig. 8c).

We found that the ethylene response related genes are specifically expressed in Jin668, including *IDD2*, *RAP2.11*, *FER*, and *PDR12*, especially, the up-regulated *AGL15 (Ghir_D08G015850)* in the xylem of Jin668 after 1 HACI, which improved the response of Jin668 hypocotyl to ethylene (Fig. 8d). In addition, wound induction can also reactivate cell proliferation. The expression of *WOX13* was induced by *WIND1*, and *WOX13* then directly up-regulated *WIND2* and *WIND3* to promote the formation of callus at wound site. Compared with TM-1, *WIND1* (*Ghir_A06G003880*) and *WOX13* (*Ghir_D08G003550*) in Jin668 was up-regulated in parenchyma cells. Besides, AP2/ERF transcription factor *PLT3* is specifically expressed in Jin668, which also contributes to the formation of callus (Fig. 8e). Then, based on the above gene regulation model of somatic embryogenesis, some core regulatory genes that involved in auxin transport, ethene response, cytokinin response, and wound induced were selected as the target gene for further functional analysis (Additional file 4: Table S3). Among which, *GhLAX1*, *GhLAX2*, and *GhLOX3* were carefully selected for CRISPR/Cas9 and overexpression experiments because of their outstanding performance in the process of cell fate transformation (Additional file 1: Fig. S10).

**Fig. 9:**
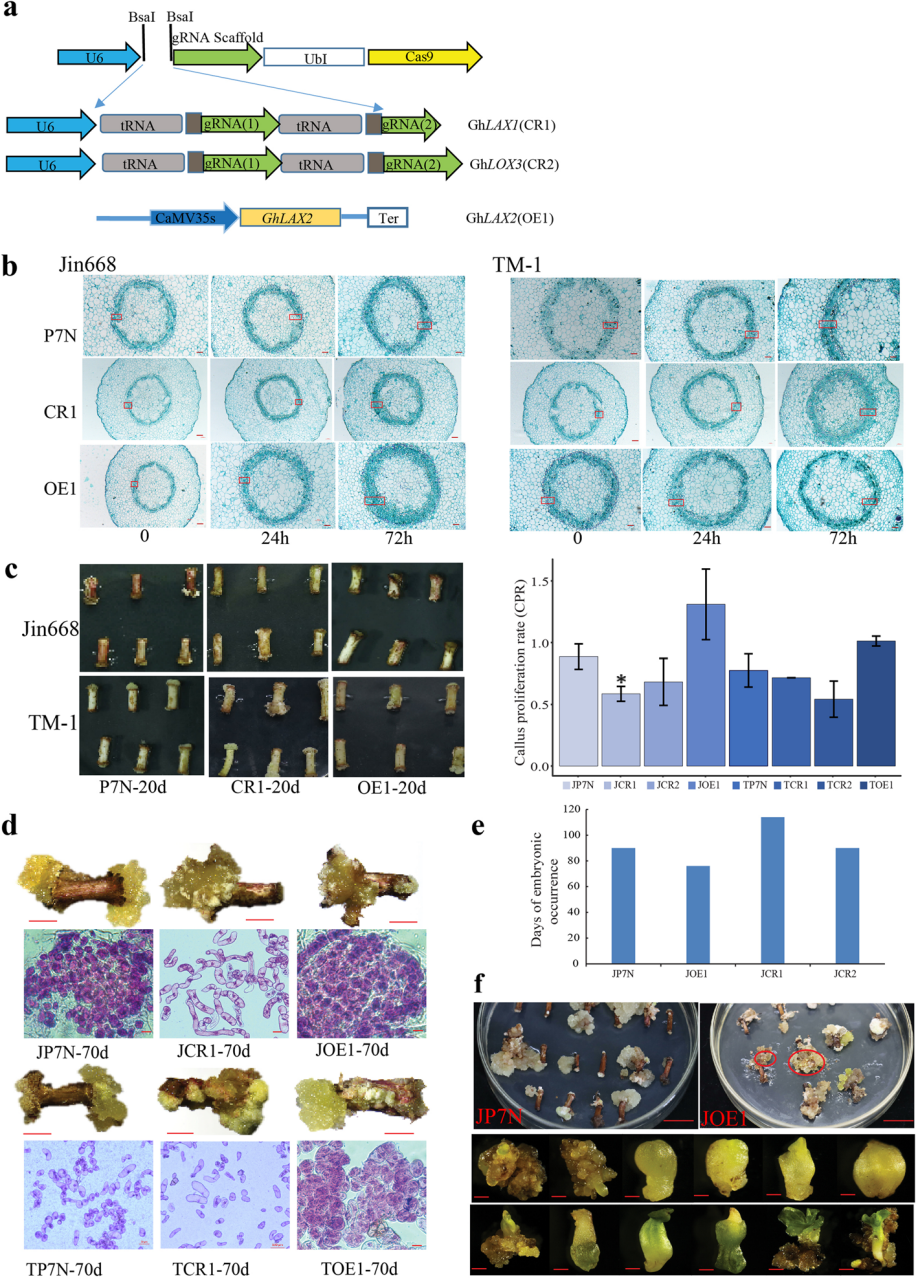
Phenotype of *GhLAX1, GhLAX2, GhLOX3* knock out and overexpression callus with hypocotyls as explants. **a** Schematic view of gRNA1, gRNA2 target sites in the *GhLAX1*, and *GhLOX3* and overexpression cassette of *GhLAX2.* **b** Paraffin sections of hypocotyls infected with *Agrobacterium* after induction on callus induction medium for 0, 24, and 72 h. The red box represents the proliferation site. **c** The phenotypes of different transgenic explants and control (P7N) at 20 days post-induction and the callus proliferation rate (CPR) of explants and control at 20 days post-induction. **d** The phenotype of callus on the *GhLAX1* knock out and *GhLAX2* overexpression explants at about 70 days post-induction. Scale bar, 100 μm. **e** Days of embryonic callus occurrence of different transgenic explants. **f** Morphology of somatic cell embryos of JOE1. Scale bar, 100 μm

In order to study the role of *AUX/LAX* in cotton regeneration, we created the CRISPR/Cas9 knockout lines of *GhLAX1* (CR1) and overexpression lines of *GhLAX2* (OE1) (Fig. 9a). After growth on callus induction medium for 3 days, both ends of hypocotyl explants began to expand (Additional file 1: Fig. S11a). Histological sections showed that the primary vascular tissue of the empty vector control tissue (pRGEB32-GhU6.7 without sgRNA, P7N) exhibited more obvious cell proliferation than the gene knockout tissues (Fig. 9b). Compared with the JP7N (Jin668 P7N control) and TP7N (TM-1 P7N control), the proliferation of primary vascular tissue cells of JCR1 (Jin668 CR1 knockout) and TCR1 (TM-1 CR1 knockout) explants was obviously repressed, and the callus from primary vascular tissue was significantly less than the control after 10 days of induction. In contrast, the overexpression explants JOE1 (Jin668 OE1) and TOE1 (TM-1 OE1) showed clear proliferation of the primary vascular tissue cells (Fig. 9b and Additional file 1: Fig. S11b). Although all explants (CRISPR and overexpression) produced callus after 20 days of induction (Fig. 9c and Additional file 1: Figure. S11a), the callus proliferation rate (CPR) after 20 days of induction showed that the CPR of JCR1 was 58% and for JOE1, 130%. JCR1 showed significantly different to control (88%) in Jin668 (t-test, *P* < 0.05), suggesting that these LAX genes may play import roles in callus proliferation in Jin668. Notably, there was no significant difference between TCR1 and TP7N (71% Vs 77%; Fig. 9c).

About 70 days after induction, TOE1 and JOE1 produced soft yellowish callus and the cells in this callus were round with dense cytoplasm, which are the typical features of embryogenic cotton callus. Compared with the control explants (JP7N), both TOE1 and JOE1 produced more embryogenic callus. However, most of the callus of JCR1 and TCR1 was hard and green, with cells of the callus having irregular shapes (elongated) with thin cytoplasm (the typical features of non-embryogenic cotton callus); this differed greatly from the TP7N control callus (Fig. 9d). At the same time, barcode test results showed that these phenotypes were caused by genome editing (Additional file 1: Fig. S11c). These results indicated that *GhLAX1* and *GhLAX2* affect the induction of callus, and the overexpression of *GhLAX2* gene promotes the formation of embryogenic callus. Next, we counted the time of somatic cell embryo formation of these transgenic materials. Compared with the control, the overexpression material can directly induce somatic cell embryos from callus in a shorter time, and the morphology of somatic embryos is normal (Fig. 9e, f).

Mechanical injury is a trigger factor for callus induction, and plant hormone JA is a key regulator for plants to respond to environmental stress. We found that *LOX3* (*Ghir_A06G021760*), encoding an enzyme in JA biosynthesis, was specifically expressed in Jin668 and not TM-1. Knockout of *GhLOX3* (CR2) in Jin668 reduced the proliferation of primary vascular tissue, but with no significant decrease in CPR compared with control P7N either in Jin668 and TM-1 (*t*-test, *P* < 0.05) (Fig. 9b, c). This means that *GhLOX3* and JA may have a small role in callus induction, while *LAX* genes have a greater effect on embryogenic potential through regulation of auxin transport.

## Discussion

### ScRNA-seq technology revealed the main cell types of cotton somatic embryogenesis

ScRNA-seq technology shows great potential and advantages in revealing cellular heterogeneity compared with traditional bulk RNA-sequencing technology. It has been used in several plant species recently, including *Arabidopsis*, maize, rice, peanut, tomato, and poplar [42, 47, 49, 67–69]. However, due to the existence of cell walls, the application of this technology still has some limitations for plants. In this study, we improved the method of protoplast isolation to obtain high-quality single cells from cotton hypocotyl for the first time. Based on this, a single-cell transcriptomic landscape of hypocotyl (the major explant for cotton tissue culture and *Agrobacterium*-mediated genetic transformation) was constructed to investigate gene expression changes linked to cell de-differentiation and proliferation under hormonal induction. Single-cell resolution gene expression landscape maps of a recalcitrant and a highly-regeneratable genotypes offer us an unique opportunity to answer questions about plant regeneration via somatic embryogenesis posed by *Science* in 2005 [70]. Compared with traditional transcriptome sequencing, single-cell transcriptome sequencing can more accurately focus on the response of different cells to external stimuli.

Our results showed that during hormone treatment associated with tissue culture, less mature cell types appear to have a higher competence for callus formation. For example, many genes involving hormone signaling and callus induction are identified in the cambium, parenchyma, and primary xylem cells of cotton hypocotyls. Callus initiation and plant regeneration is affected by many factors, including the hormones auxins, cytokinin, and ethylene, and mechanical tissue wounding. By comparing the single-cell transcriptome landscape between the highly regenerable genotype Jin668 and recalcitrant genotype TM-1, it was found that the cambium and primary xylem cells of Jin668 and TM-1 exhibit distinct gene expression profiles. For example, *WIND1* was found mainly expressed in the primary xylem of cotton hypocotyl and decreased in TM-1, but increased in Jin668. Compared to TM-1, auxin polar transport and signal transduction genes *Ghir_D04G006710*, *Ghir_D11G011100*, *Ghir_D01G000880*, *Ghir_A04G012980*, *Ghir_A11G000170*, *Ghir_D04G009130*, *Ghir_D06G012360*, and *PLT3* (*Ghir_D10G010630*) were specifically highly expressed in Jin668, associated with callus induction and plant regeneration. Interestingly, some transcription factors involved in cell fate determination in *Arabidopsis*, such as *BBM* and *WUS*, have also been reported to improve somatic embryogenesis in monocotyledon plants such as maize, rice, and wheat [71–73]. However, they were not significantly expressed both either Jin668 or TM-1. These transcription factors may not therefore play important roles in the regeneration of cotton.

### Auxin plays an important role in cotton somatic embryogenesis

Auxin plays a key role in cell proliferation and the establishment of embryo polarity during both zygotic and somatic embryogenesis. Studies have shown that auxin-dependent cellular specification during embryo development requires balanced auxin transport, involving both influx and efflux mechanisms, and this transport is maintained through positive transcriptional feedback of auxin signaling [74]. Pseudo time analysis found that the auxin efflux carrier gene *PIN1* was distributed along the cambium/parenchyma and primary xylem branch of Jin668 and TM-1. Compared to TM-1, the auxin influx carrier genes *LAX1* and *LAX2* were specifically expressed in the cambium of Jin668, while other auxin transport genes, such as *PLC2* (*Ghir_D04G007290*), *PIS1* (*Ghir_D11G011100* and *Ghir_D04G009130*), and *CSEF* (*Ghir_A11G000170*) showed reduced expression in Jin668. The analysis of co-expression networks also revealed that TM-1 has a different auxin regulatory network compared to Jin668. Therefore, we speculate that one major reason why TM-1 is recalcitrant during tissue culture and plant regeneration is that the establishment of embryonic callus does not occur, and this is likely dependent to a significant extent on auxin homeostasis in those cells. Organogenesis is another major way of plant regeneration in many plant species such as tobacco, wheat, and *Arabidopsis thaliana*. Previous studies have reported that the callus of *Arabidopsis* roots, leaves, and petals was derived from pericycle-like cells, which was similar to the process of lateral root initiation [37, 75]. Our results show that several genes related to lateral root initiation, including *IAA14*, *IAA22,* were expressed in the primary xylem of cotton. However, these genes showed a downward expression trend with time in tissue culture, which may be the reason why the somatic embryogenesis is the major pathway for cotton plant regeneration rather than organogenesis, at least under the hormonal conditions applied.

### By comparing the differences between cotton with different regeneration abilities reveals the regulatory network of somatic embryogenesis

Together, our results indicate that auxin, cytokinin, ethylene, and wounding-related genes have different expression patterns between Jin668 and TM-1 (Fig. 8). The ethylene responsive gene *AGL15* affects callus induction during somatic embryogenesis by affecting the ratio of GA/ABA [5, 11]. Wound-inducing factor *WIND1* plays a positive role in regulating callus induction, and this gene is up-regulated in Jin668. The auxin transport-related genes are significantly different between regenerable and non-regenerable explant tissues. The auxin influx-related genes *LAX2* and *LAX1* may affect the distribution of auxin and thus affect somatic embryogenesis by acting on the auxin polar transport gene *PIN1* and other auxin-regulated pathways. The role of the *LAX1* and *LAX2* genes in embryogenic callus formation was confirmed here experimentally.

In conclusion, to verify the effect of these genes on callus induction and somatic embryogenesis of cotton, CRISPR/Cas9 knock out and overexpression were performed for three key genes identified by scRNA-seq technology described previously. In Jin668, the callus proliferation rate was decreased compared with the control after knockout of auxin-related genes *LAX1* and *LAX2* and JA-related genes *LOX3*. Meanwhile, when the gene Gh*LAX2* was overexpressed in the TM-1, it was found to promote the cell proliferation of primary vascular tissue and formation of embryogenic callus. These results suggest that the somatic embryogenesis-related genes resolved at the single-cell level have the potential to alter the regenerative capacity of recalcitrant genotype materials, thereby may break the genotype dependence of somatic embryogenesis in cotton. The study illustrates how scRNA-seq can better define the anatomy of plant cell heterogeneity and the analysis of developmental trajectories. The discovered cell clusters, and the cluster-specific marker genes, lay the foundation for our analysis of the developmental and physiological functions of these cell types, providing opportunities to study callus induction and somatic embryogenesis in other species. Through the combination of identification of a large number of candidate genes and subsequent gene function analysis, we can expect to find the key genes that determine the regeneration ability of cotton and provide new genetic resources for optimizing the existing regeneration system and breaking the genotype restriction for plant regeneration through somatic embryogenesis.

## Conclusions

In general, the successful application of scRNA-seq technology provides us with an opportunity to understand the molecular mechanism of somatic embryogenesis at the single-cell level. In our work, we analyzed the difference between regenerable and recalcitrant materials in somatic embryogenesis. The callus of cotton hypocotyl mainly comes from primary vascular tissue cells. Auxin is the main factor affecting cell dedifferentiation, and the polar transport of auxin determines the ability of cells to obtain embryos. In addition, cell communication between different cell types also affects cell dedifferentiation. During this process, some decisive genes affect the induction of callus and the formation of somatic embryos. In addition, our data reliability is also verified by knockout and overexpression experiments. In the future, we will focus on analyzing the regulatory mechanism of these predicted genes related to somatic embryogenesis, more comprehensively study the mechanism behind somatic embryogenesis, and break the restriction of genotype to achieve more variety regeneration.

## Methods

### Plant materials and prepare for single-cell suspension

All the tissue culture and callus induction and proliferation experiments of upland cotton (*Gossypium hirsutum* L.) Texas Marker-1 (TM-1) and Jin668 [6] were performed at Huazhong Agricultural University (Wuhan, Hubei, China) following our previous report [9, 76]. The hypocotyls of etiolated seedlings were cut into 5–7 mm sections and used as explants for callus induction on MSB (2,4-D) medium. The single-cell suspension from explants were harvested at 0, 1, 6, and 12 HACI with two replicates. In brief, the hypocotyl was chopped into small discs with scalpel blade and immersed in 3–5 ml RNase-free enzyme solution (1.5% cellulase R10, 1.5% macerozyme R10, 0.4M mannitol, 10mM KCl, 10mM CaCl_2_, and 0.1% BSA). Then digested for 4 h with gentle shaking (30 r/min) in the dark at room temperature. The protoplasts were filtered with cell strainer (40 μm diameter, Falcon #352,340) and transferred to a centrifuge tube containing phosphate-buffered saline (PBS) (with 0.04% BSA), then slightly mix and centrifuge (4℃, 70 g and 2 min), and suspended with PBS as above described. After performing the second filtration with a cell strainer, the single-cell suspension was stained with fluorescein diacetate (FDA) at 14 mM final concentration for 3 min on ice and the number and activity of cells was calculated with microscope (ZEISS Axioscope A1). The protoplasts with a concentration of 1500–2000 cells/ml were then processed with the 10 × Genomics Single Cell Protocol (CG00052, RevC).

### Single-cell RNA-seq library construction and sequencing

Approximately, 15,000 counted cells were used for reverse transcription and subsequently library preparation according to instruction in the 10 × Chromium Single Cell 3’ Reagent Kits v3. The qualified libraries were then pooled and paired-end sequenced across six lanes on a HiSeq2500. In total, more than 51,551,182 reads for each library were obtained.

### Bulk RNA-seq and analysis

Bulk RNA-seq from Jin668 and TM-1 were applied to RNA extracted from dissociated and un-dissociated hypocotyl and sequenced on the HiSeq X Ten platform. Sequence reads were trimmed using Trimmomatic (v0.39) [77] and aligned to the TM-1 genome [78] (http://cotton.hzau.edu.cn/EN/Download.htm) with HISAT2 (v2.2.1) [79]. Gene expression values were calculated using StringTie (v2.1.4) [80], and DEseq2 (v1.4.5) [81] was used to calculate differentially expression (fold change > 2 and *P* < 0.01).

### Single-cell RNA-seq read processing and cell clustering

The raw single-cell RNA-seq dataset was performed demultiplexing, barcode assignment, and UMI quantification with aligned to reference-grade TM-1 genome [78] (http://cotton.hzau.edu.cn/EN/Download.htm) using the Cell Ranger Software Suite (v4.0.0). A total of 91.3% mapping rate and more than 3000 cells for each sample was captured. All feature-barcode matrices of samples from different induced time points (0, 1, 6, and 12 HACI) for both TM-1 and Jin668 with two biological repetitions were then aggregated using “cellranger aggr” with normalize set to “mapped”.

The aggregated matrix data was imported into R using Seurat (v3.2.3) and scater (version 1.10.1) package for further data analysis [82]. Outlier cell with library size and number of expressed genes more than 3 median absolute deviations (MADs) below the median value, unique molecular identifiers (UMIs) counts under 10,000 or greater than 500, and detected genes under 4100 or greater than 200 were removed. Low abundance genes with detected less than 10 cells and protoplasting-induced genes during enzymatic digestion for generating protoplasts (detected by bulk RNA-seq) were also removed. Potential doublets were identified using the DoubletFinder algorithm (version 2.0.2) [83] with three input parameters: the number of expected real doublets (nExp) (cell numbers/100000), the number of artificial doublets (pN) (pN = 0.25), and the neighborhood size (pK) (optimal pK value was inferred by paramSweep_v3, summarizeSweep and find.pK functions in turn). Then, NormalizeData with LogNormalize method transformation and SCTransform functions were used to normalize and identify the highest expression variability genes. Top 3000 highly variable genes were used for principal component analysis (PCA) dimensionality reduction and first 20 PCs were selected according to combined with PCA elbow plot and DimHeatmap function. Clustering analysis was performed at a resolution parameter of 0.4 and 0.2 for TM-1 and Jin668, respectively. The *t*-SNE [84] and UMAP [85] methods were used to visualize the cell clusters. Identification of all markers for each cluster were performed using function FindAllMarkers with a Wilcoxon rank sum test and subsequently classification of cluster using marker orthologs genes mapped from *Arabidopsis thaliana* (Additional file 4: Table S3), which was collected from Plant Single Cell Transcriptome Hub (PsctH; http://jinlab.hzau.edu.cn/PsctH/) [51] and literature review.

### *RNA *in situ* hybridization*

The dehydration and infiltration of the hypocotyl were performed in ethanol and paraffin series as in a previous study [86] and then the section was stored at 4°C. We selected specifically expressed marker genes from each cell cluster. The specific regions of marker genes were cloned into the pGEM-TEasy (TRANDGEN BIOTECH), then Digoxigenin RNA labeling kit (Roche) was used for in vitro transcription and labeling. The hybridization and immunological detection were performed as described previously [87], and microscopy was carried out in bright-field mode using Leica DM6 B. The primers were listed in Additional file 3: Table S2.

### Single-cell pseudotime trajectory analysis

Trajectory inference and pseudo time analysis was performed using Monocle2 (v2.8.0) [88]. The clustered Seurat object was import Monocle2 and differentialGeneTest function with CellType as full model in differential expression tests (fullModelFormulaStr) was used to calculate the variance in each gene’s expression across cells. The chosen variable genes were used to define a developmental trajectory. Then, we perform the dimension reduction (reduce Dimension function with max_components = 2 and method = ‘DDRTree’) and trajectory analysis (orderCells funvtion with default parameters). The cell trajectory was plotted by “plot_cell_trajectory” with coloring by Pseudotime and CellType. Genes dynamically expressed along the pseudotime were also clustered and visualized using the plot_pseudo time_heatmap function. Branch expression analysis modeling (BEAM) was used to analyze the pseudo-time-dependent or branch-dependent genes. The genes that were significantly branch-dependent were visualized by the “plot_genes_branched_heatmap” function.

### RNA velocity analysis

RNA velocity was calculated by velocyto package based on a previous publication ( http://velocyto.org/) [64]. Briefly, expression matrix of unspliced and spliced mRNA in each sample for both Jin668 and TM-1 were generated with velocyto CLI (v.0.17.17) with default parameters. The output loom files of each HACI were combined using “loompy” and then imported into velocyto.R (v.0.6) for the downstream analysis. The unspliced and spliced counts of the cells from primary xylem, parenchyma, and cambium were extracted. To filter out lowly variable genes, data normalization and variable gene detection were performed. The final filtered genes were used to estimate RNA velocity with “gene.relative.velocity.estimates” function (kCells = 20). In addition, we also computed fate probabilities and summarized them in a fate map and also visualized with pie charts on a directed implementation of partition-based graph abstraction (PAGA) [89].

### Differential gene expression analysis

Differential gene expression analysis was performed based on the tutorial in Harvard Chan Bioinformatics Core (HBC) (https://hbctraining.github.io/scRNA-seq/lessons/pseudobulk_DESeq2_scrnaseq.html). Briefly, the preprocessed, normalized and clustered scRNA-seq data from Seurat workflow was used to build SingleCellExperiment object. Then sample-level metadata and counts were aggregated for each sample within each cell type in TM-1 and Jin668, respectively. Next, DESeqDataSetFromMatrix and DESeq functions in DESeq2 [81] were used to perform create of DESeq2 object and differential expression analysis. Genes with adjusted *P* < 0.05 for each cell-type pair were labeled as significantly differentially expressed.

### Venny diagram

Venny (https://bioinfogp.cnb.csic.es/tools/venny/index.html) was applied to analysis genes which were specially expressed in cambium and primary xylem cells of Jin668.

### Analysis of cell communication and regulatory network

To understand the co-expression relationships between genes at a systems level, weighted gene co-expression network analysis (WGCNA) [90] were utilized to inference gene regulatory network-related to somatic embryogenesis. WGCNA was performed on normalized gene expression data that from 3000 highly variably expressed genes determined by FindVariableGenes function in Seurat. Briefly, we first aggregated data from 10 cells in the same cluster to make pseudo-cells for each cell type. Then R package WGCNA used to constructs a gene co-expression matrix and groups of closely co-expressed genes into modules. Hierarchical clustering of modules is displayed as topological overlap matrix (TOM) plots and similarity between gene modules are displayed as adjacency plots. The hub genes for each module were identified as module eigengene based connectivity kME > 0.8 and *P* < 0.05.

### Gene enrichment analysis

The cluster-enriched genes and differently expressed genes were feed into clusterProfiler [91] for GO and KEGG enrichment analysis. All genes in the *Gossypium hirsutum* genome or all genes expressed in corresponding cell types were used as a background. Fisher’s exact test was used and the false discovery rate (FDR) was calculated for multiple test correction. Only less than 0.05 (FDR < 0.05) were retained. The complete annotation dataset for biological process, molecular function, and cellular component GO terms was used for analysis.

### CRISPR-Cas9 and overexpression analysis for candidate genes related to cotton plant regeneration

Three genes identified from the scRNA-seq and regulatory network relates to somatic embryogenesis were selected to confirm their function by CRISPR/Cas9 and overexpression technologies. The sgRNAs of all candidate genes were designed using CRISPR [92] program with *Gossypium hirsutum* genome as reference. Each sgRNA was cloned into CRISPR/Cas9 vector pRGEB32-GhU6.7 and introduced into *Agrobacterium* strain GV3101 by electroporation and then carry out genetic transformation via *Agrobacterium*-mediated with Jin668 and TM-1 as transformation receptor according to our previous publications [6, 8, 93, 94].

The full-length coding sequence of *GhLAX2* was amplified from cDNA and cloned into the overexpress vector PK2GW7 to construct the vector *35S*::*GhLAX2* by Gateway Technology (Invitrogen, Carlsbad, CA). Both Jin668 and TM-1 were used for transformation receptor via *Agrobacterium* mediated genetic transformation as described previously.

The callus increment rate (CPR) was calculated as the fold change in weight gained of explants at 20 days post-induction. Three biological replicates were included and each replicate represented at least five culture dishes with more than 20 explants per dish. All statistical analysis were performed with R (Version 4.0.0) (http://www.R-project.org/). In the two-sided test, *P* < 0.05 was considered as being statistically significant.

### Website for accessibility and visualization of data

To allow optimal accessibility of this cotton hypocotyl cells scRNA-seq dataset by the scientific community, we construction the Cell Landscape in Cotton (CLC, http://jinlab.hzau.edu.cn/CLC/ or http://jinlab.hzau.edu.cn:8000/CLC/) website that used a bootstrap framework to improve overall adaptability and interactivity and used Python and dash framework to preprocessing and visualization of back-end data. The main functions of the CLC website are divided into search and download. The cell clusters, cell type-specific expression patterns of individual genes, expression levels along developmental trajectories, and raw scRNA-seq data downloads can be easy accessed.

### Supplementary Information

**Additional file 1: Fig. S1.** Single cell sampling of cotton hypocotyl and the number of cells obtained. **Fig. S2.** Single cell map of cotton hypocotyl. **Fig. S3.** UMAP visualization shows these cotton hypocotyl cells in different time points both in Jin668 and TM-1. **Fig. S4.** Expression profiles of marker and SE related genes. **Fig. S5.** Genes expressed in primary vascular tissue cells of Jin668 and TM-1. **Fig. S6.** Pseudotime trajectory of primary xylem of Jin668. **Fig. S7.** The fate maps of directional aggregation and gene expression trend of local cell clusters. **Fig. S8.** Network analysis SE-related genes of Jin668 and TM-1 hypocotyl primary vascular cells. **Fig. S9.** Gene regulation and expression modules in different cell types. **Fig. S10.** The expression patterns of selected SE related genes in Jin668 (left) and TM-1 (right). **Fig. S11.** Phenotype of knock out with hypocotyls as explants.

**Additional file 2: Table S1. **Number of cells identified in our single cell data. The same cell types of Jin668 and TM-1 were aligned one by one.

**Additional file 3: Table S2. **Cell quality of 16 scRNA-seq samples and primer information used in the experiment.

**Additional file 4: Table S3 **Summary of marker genes of different cell types in Jin668 and TM-1. The differential genes obtained through Venn diagram and pseudo-time analysis and the final selected genes related to somatic embryogenesis.

**Additional file 5: Table S4. **Differentiated genes expressed in epidermis, cortex, primary xylem, parenchyma cell, cambium, xylem vessel and phloem of Jin668 and TM-1 at different time points.

**Additional file 6. **Review history.

## Data Availability

The authors declare that all data supporting the findings of this study are available within the article and its supplementary information files or from the corresponding author upon reasonable request. The raw data of single-cell RNA-seq and bulk RNA-seq for Jin668 and TM-1 have been deposited in the Sequence Read Archive under accession number PRJNA895968 [95] and PRJNA895970 [96]. Data can also be explored at http://jinlab.hzau.edu.cn/CLC/ or http://jinlab.hzau.edu.cn:8000/CLC/. The microscope data generated in this study have also been deposited in a public repository and can be explored at https://figshare.com/projects/Single_cell_landscape_of_cotton_hypocotyl/172416 [97]. There were no custom scripts and software was used other than those mentioned in the “ Methods” section.
