# Author Correction: Single-cell resolution analysis reveals the preparation for reprogramming the fate of stem cell niche in cotton lateral meristem

**DOI:** 10.1186/s13059-023-03051-3

**Published:** 2023-09-18

**Authors:** Xiangqian Zhu, Zhongping Xu, Guanying Wang, Yulong Cong, Lu Yu, Ruoyu Jia, Yuan Qin, Guangyu Zhang, Bo Li, Daojun Yuan, Lili Tu, Xiyan Yang, Keith Lindsey, Xianlong Zhang, Shuangxia Jin

**Affiliations:** 1 Hubei Hongshan Laboratory, National Key Laboratory of Crop Genetic Improvement, Huazhong Agricultural University, Wuhan, 430070 Hubei China; 2 Xinjiang Key Laboratory of Crop Biotechnology, Institute of Nuclear and Biological Technology, Xinjiang Academy of Agricultural Sciences, Wulumuqi, 830000 Xinjiang China; 3 Department of Biosciences, Durham University, Durham, DH1 3LE UK

**Correction: Genome Biol 24, 194 (2023)**

**https://doi.org/10.1186/s13059-023-03032-6**

Following publication of the original article [1], the authors reported an error in Fig. 9, namely a missing significant difference symbol for JCR1 and a redundant significant difference symbol for JOE1. The updated Fig. 9 is available in this Correction.

**Fig. 9:**
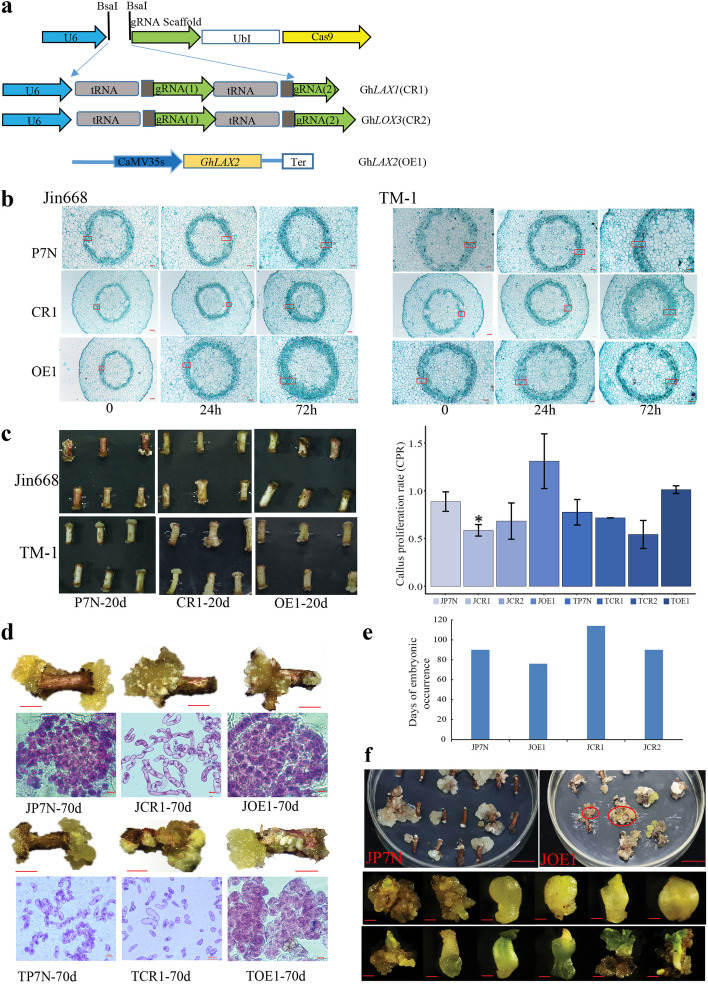
Phenotype of *GhLAX1*, *GhLAX2*, *GhLOX3* knock out and overexpression callus with hypocotyls as explants. a Schematic view of gRNA1, gRNA2 target sites in the *GhLAX1*, and *GhLOX3 *and overexpression cassette of *GhLAX2*. b Paraffin sections of hypocotyls infected with *Agrobacterium* after induction on callus induction medium for 0, 24, and 72 h. The red box represents the proliferation site. c The phenotypes of different transgenic explants and control (P7N) at 20 days post-induction and the callus proliferation rate (CPR) of explants and control at 20 days post-induction. d The phenotype of callus on the *GhLAX1* knock out and *GhLAX2* overexpression explants at about 70 days post-induction. Scale bar, 100 μm. e Days of embryonic callus occurrence of diferent transgenic explants. f Morphology of somatic cell embryos of JOE1. Scale bar, 100 μm

Additionally, the following text describing the experimental results shown in Fig. 9 has been amended as follows:

**Previous text:** Although all explants (CRISPR and overexpression) produced callus after 20 days of induction (Fig. 9c and Additional file 1: Figure. S11a), the callus proliferation rate (CPR) after 20 days of induction showed that the CPR of JCR1 was 58% and for JOE1, 130%, both significantly different to control (88%) in Jin668 (t-test, *P* < 0.05), suggesting that these *LAX* genes may play import roles in callus proliferation in Jin668. Notably, there was no significant difference between TCR1 and TP7N (71% Vs 77%; Fig. 9c).

**Updated text:** Although all explants (CRISPR and overexpression) produced callus after 20 days of induction (Fig. 9c and Additional file 1: Fig. S11a), the callus proliferation rate (CPR) after 20 days of induction showed that the CPR of JCR1 was 58% and for JOE1, 130%. JCR1 showed significantly different to control (88%) in Jin668 (t-test, *P* < 0.05), suggesting that these *LAX* genes may play import roles in callus proliferation in Jin668. Notably, there was no significant difference between TCR1 and TP7N (71% Vs 77%; Fig. 9c).

The original article has been updated.
