# Supplementary material for: Single-cell resolution analysis reveals the preparation for reprogramming the fate of stem cell niche in cotton lateral meristem
Source: Genome Biol. 2023 Aug 25;24:194. doi: 10.1186/s13059-023-03032-6 (PMC10463415; doi:10.1186/s13059-023-03032-6)
Supplement: Supplementary file 1 — Additional file 1: Fig. S1. Single cell sampling of cotton hypocotyl and the number of cells obtained. Fig. S2. Single cell map of cotton hypocotyl. Fig. S3. UMAP visualization shows these cotton hypocotyl cells in different time points both in Jin668 and TM-1. Fig. S4. Expression profiles of marker and SE related genes. Fig. S5. Genes expressed in primary vascular tissue cells of Jin668 and TM-1. Fig. S6. Pseudotime trajectory of primary xylem of Jin668. Fig. S7. The fate maps of directional aggregation and gene expression trend of local cell clusters. Fig. S8. Network analysis SE-related genes of Jin668 and TM-1 hypocotyl primary vascular cells. Fig. S9. Gene regulation and expression modules in different cell types. Fig. S10. The expression patterns of selected SE related genes in Jin668 (left) and TM-1 (right). Fig. S11. Phenotype of knock out with hypocotyls as explants. [file 13059_2023_3032_MOESM1_ESM.docx]

**Single-cell resolution analysis reveals the preparation for reprogramming the fate of stem cell niche in cotton lateral meristem**

**Additional file 1:** Supplementary Figures S1-S11.

**
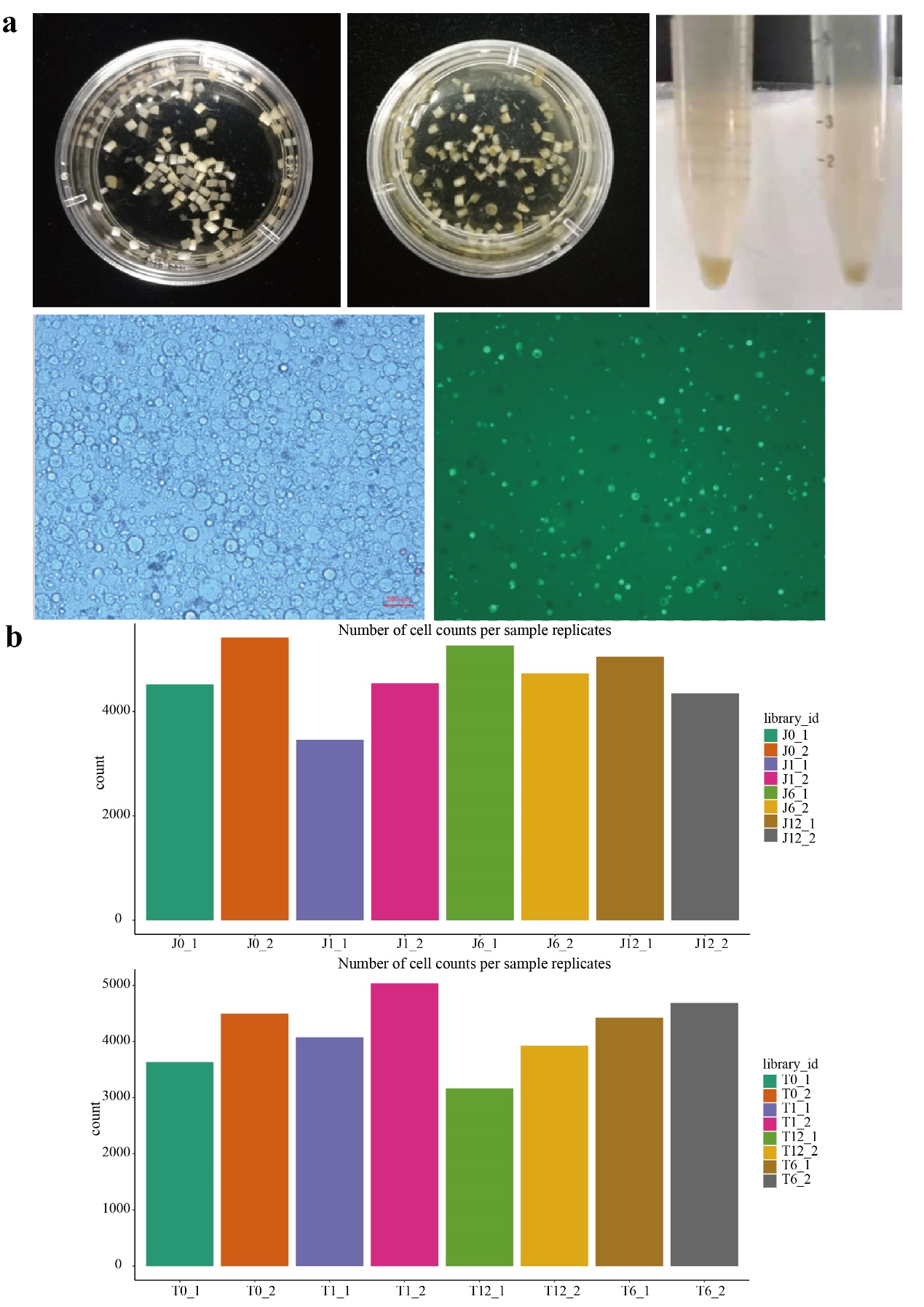
**

**Fig. S1. Single cell sampling of cotton hypocotyl and the number of cells obtained.** (**a**) Single cells of cotton hypocotyl obtained by enzymatic hydrolysis. The living cells were stained with FDA. Scale bar, 100 μm. (**b**) Number of cells (before filtering) measured at different time points of induction. Two repeats at each time point.

**
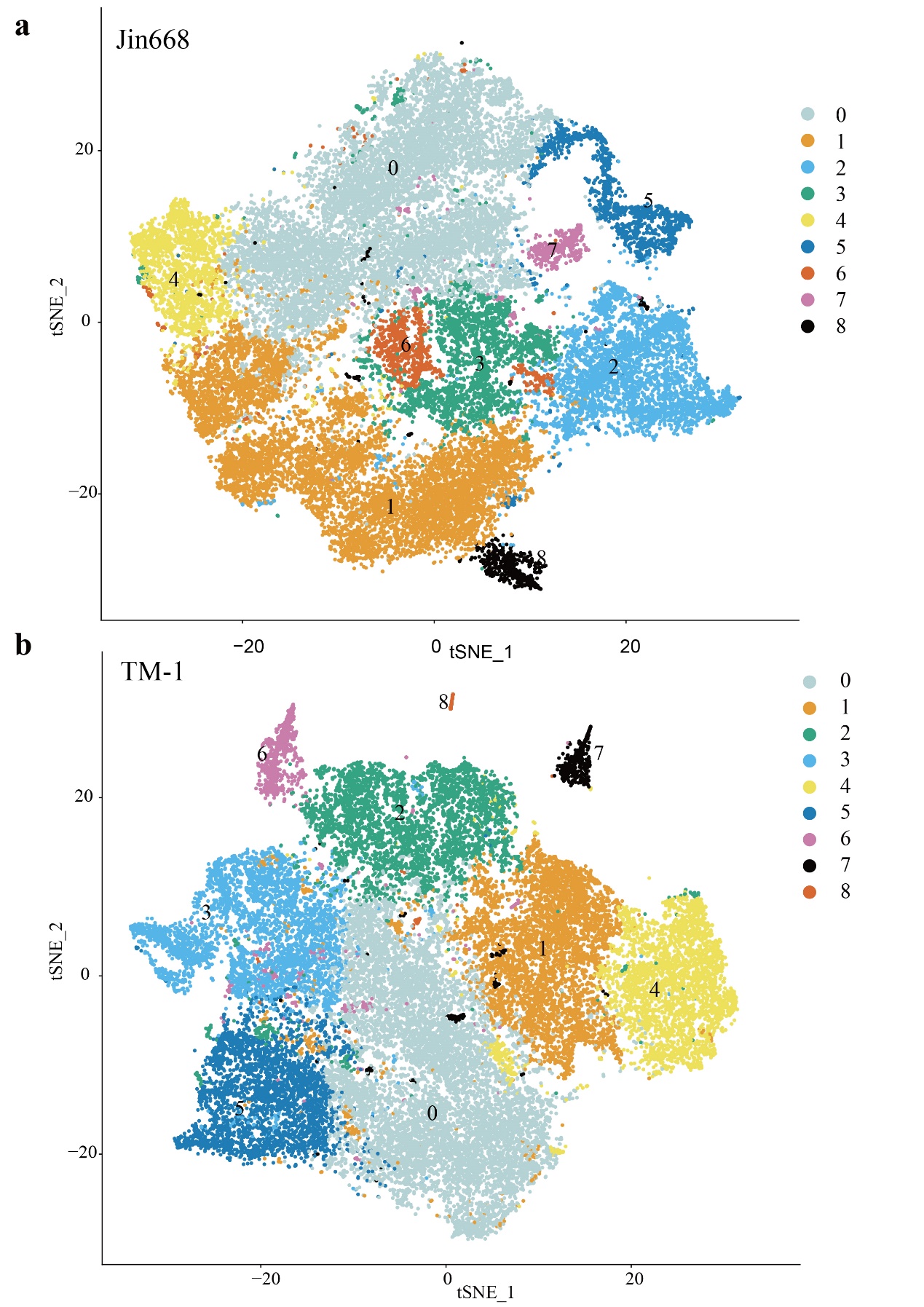
Fig. S2.** **Single cell map of cotton hypocotyl.** (**a**) *t*-SNE visualization cotton hypocotyl cells were grouped into 9 clusters in Jin668. (**b**) *t*-SNE visualization cotton hypocotyl cells were grouped into 9 clusters in TM-1. Different colors represents different cell types.

**
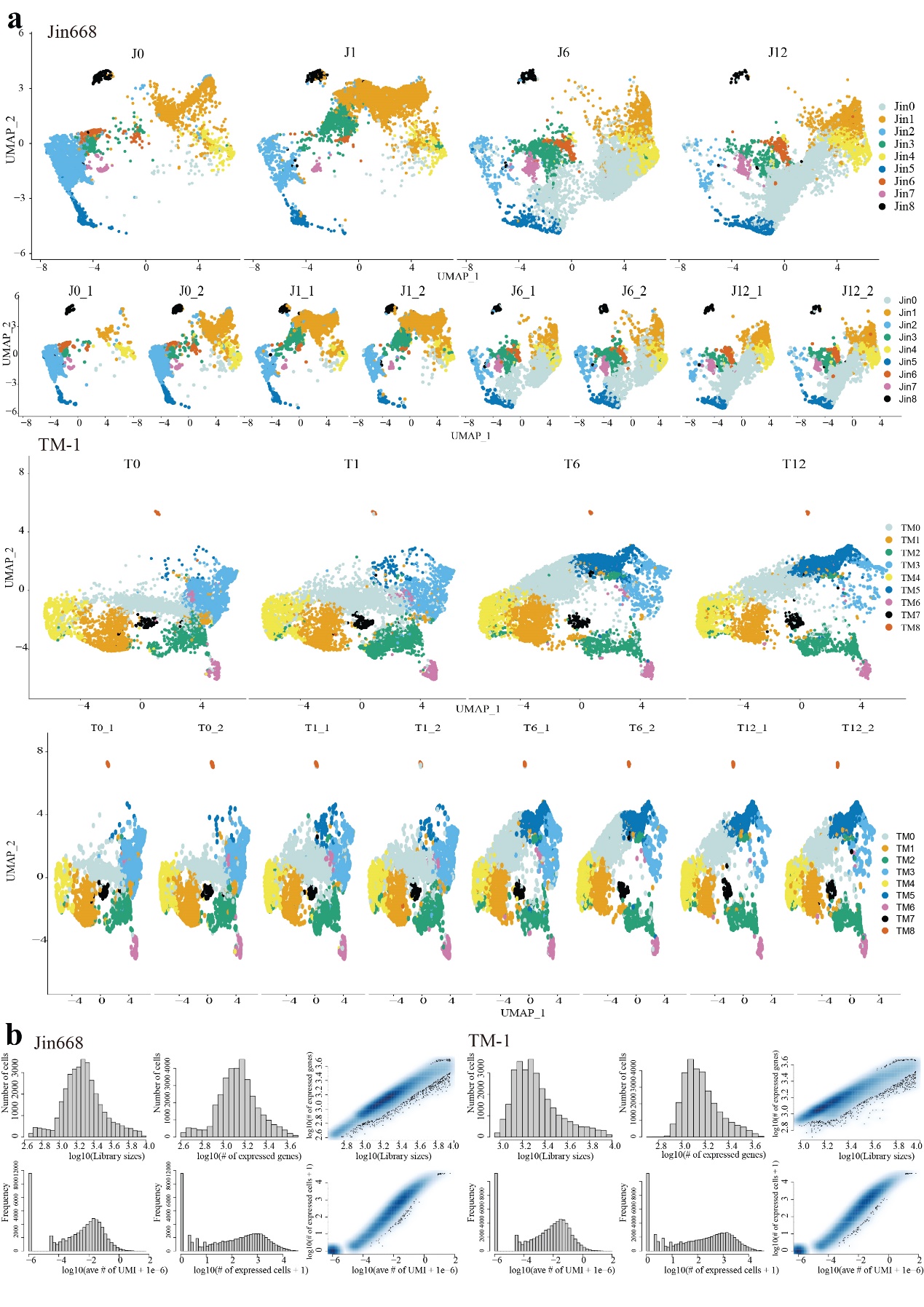
**

**Fig. S3. UMAP visualization shows these cotton hypocotyl cells in different time points both in Jin668 and TM-1. (a)** Single cell maps of Jin668 and TM-1 at different time points and different reduplicates. Different colors represents different cell types. **(b)** Distrubition of cell numbers and mRNA profiles in Jin668 and TM-1.
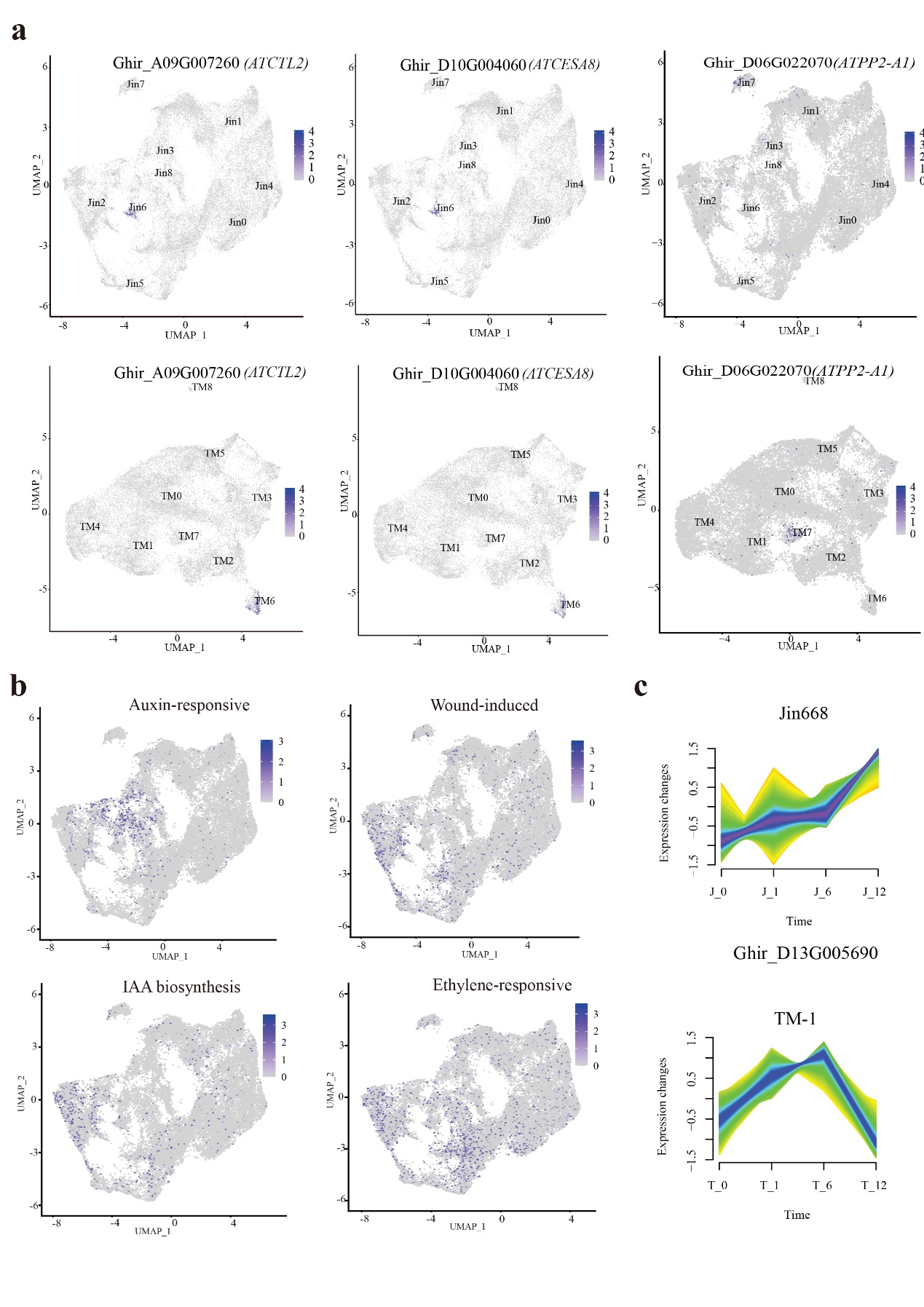

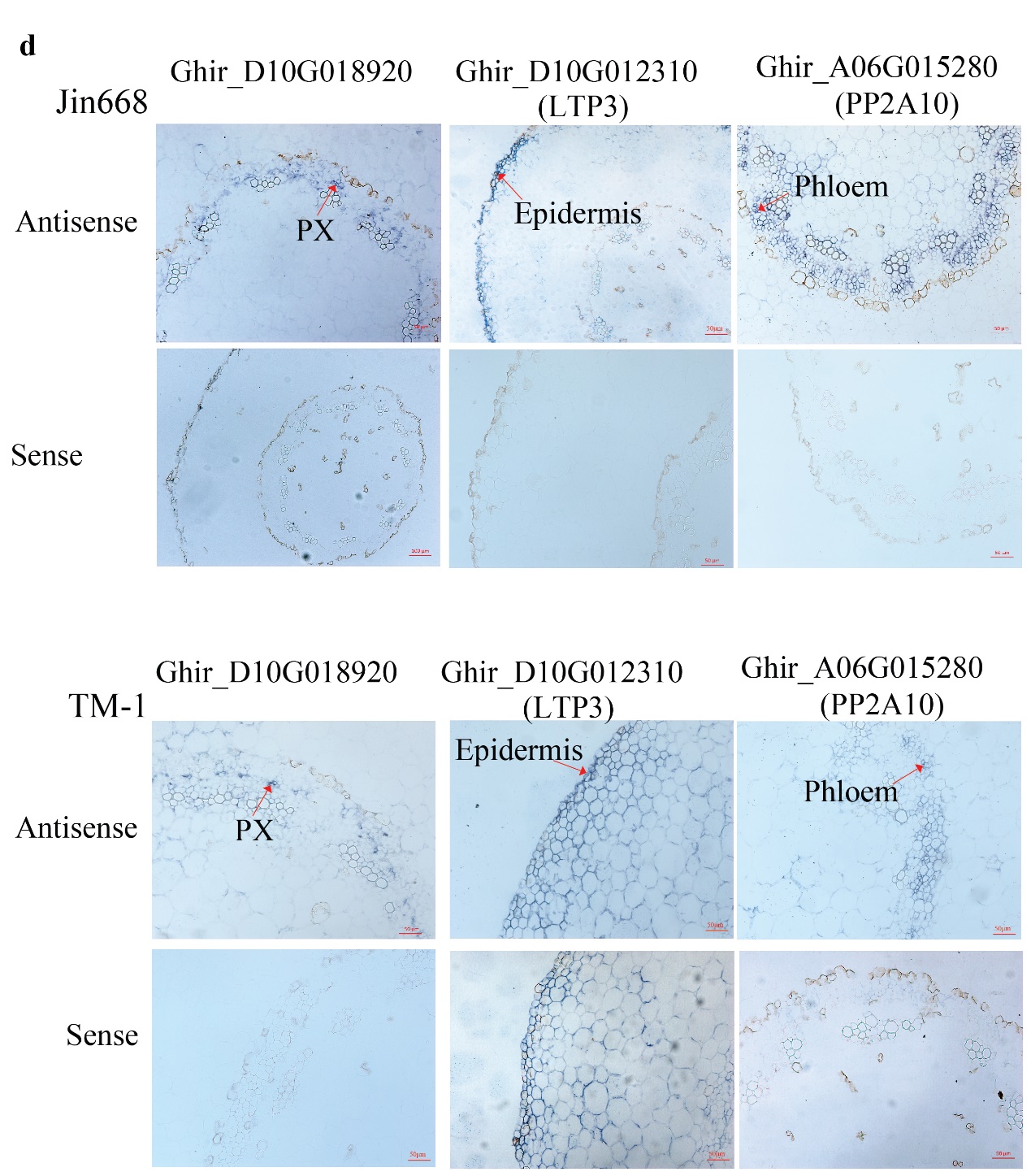


**
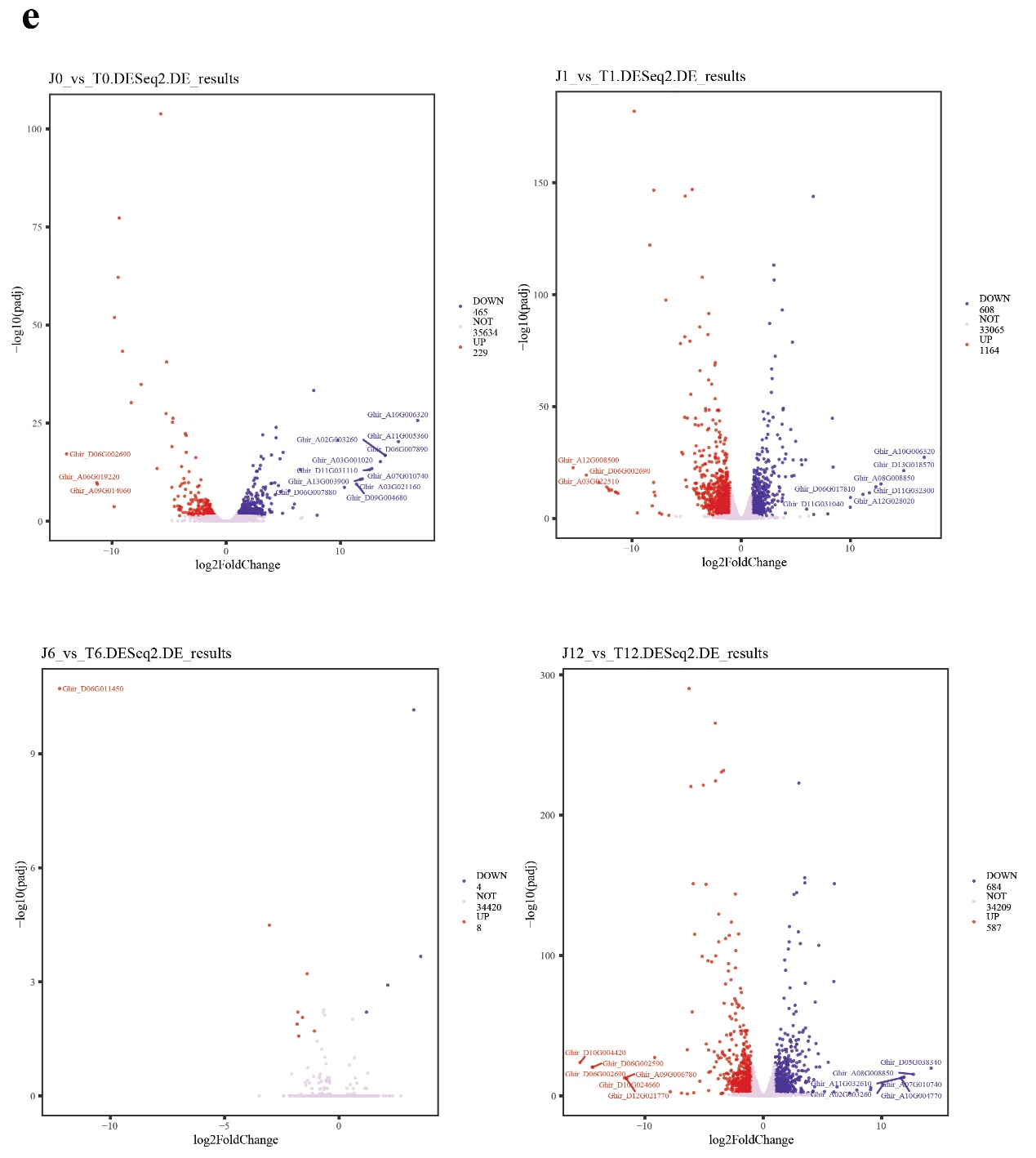
**

**Fig. S4. Expression profiles of marker and SE related genes. (a)** Distribution feature of cluster marker genes on UMAP plot. **(b)** Distribution feature of genes related to SE in Jin668, including auxin, wound and ethylene. **(c)** Expression patterns of *SERK1* in the primary xylem of Jin668 and TM-1. Color bar indicates the relative expression level.

**(d)** RNA *in situ* hybridization of marker genes in Jin668 and TM-1. Scale bar, 50 μm. **(e)** Differential expression gene (DEGs) analysis between Jin668 and TM-1 after induction at the same time with traditional bulk-RNA-seq-method. Colored by fold−change direction (padj <= 0.05 & log2FoldChange >= 1).

**
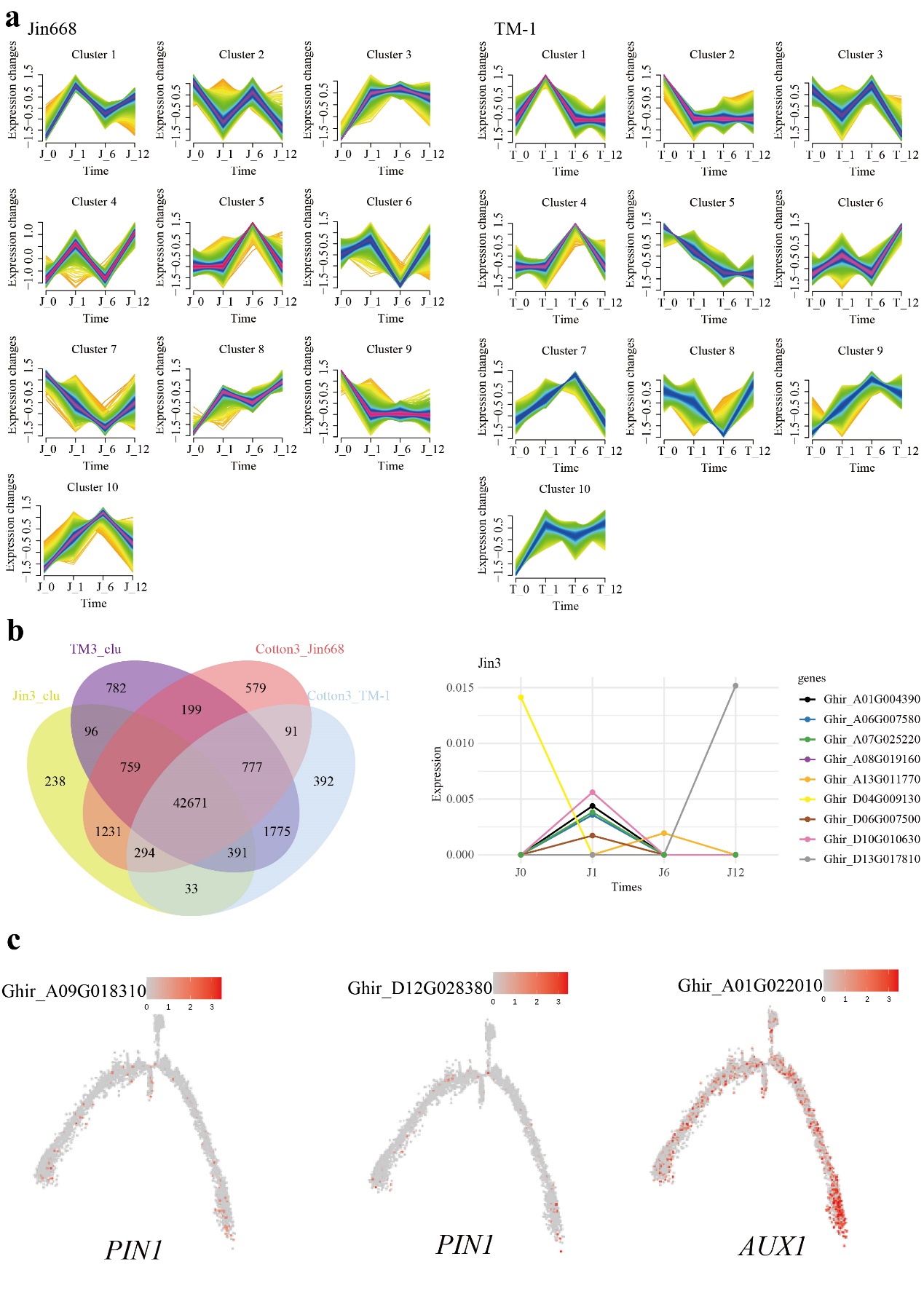
Fig. S5. Genes expressed in primary vascular tissue cells of Jin668 and TM-1. (a)** Clustered gene expression patterns in parenchyma cells of Jin668 and TM-1 at different induction times. **(b)** Venn digram showing genes specifically expressed in the parenchyma cells of Jin668 (left) and the expression trend of auxin transport related genes after induction at different times (right). **(c)** Expression patterns of representative auxin-related genes (*PIN1* and *AUX1*) are shown over the course of pseudotime. Color bar indicates the relative expression level.


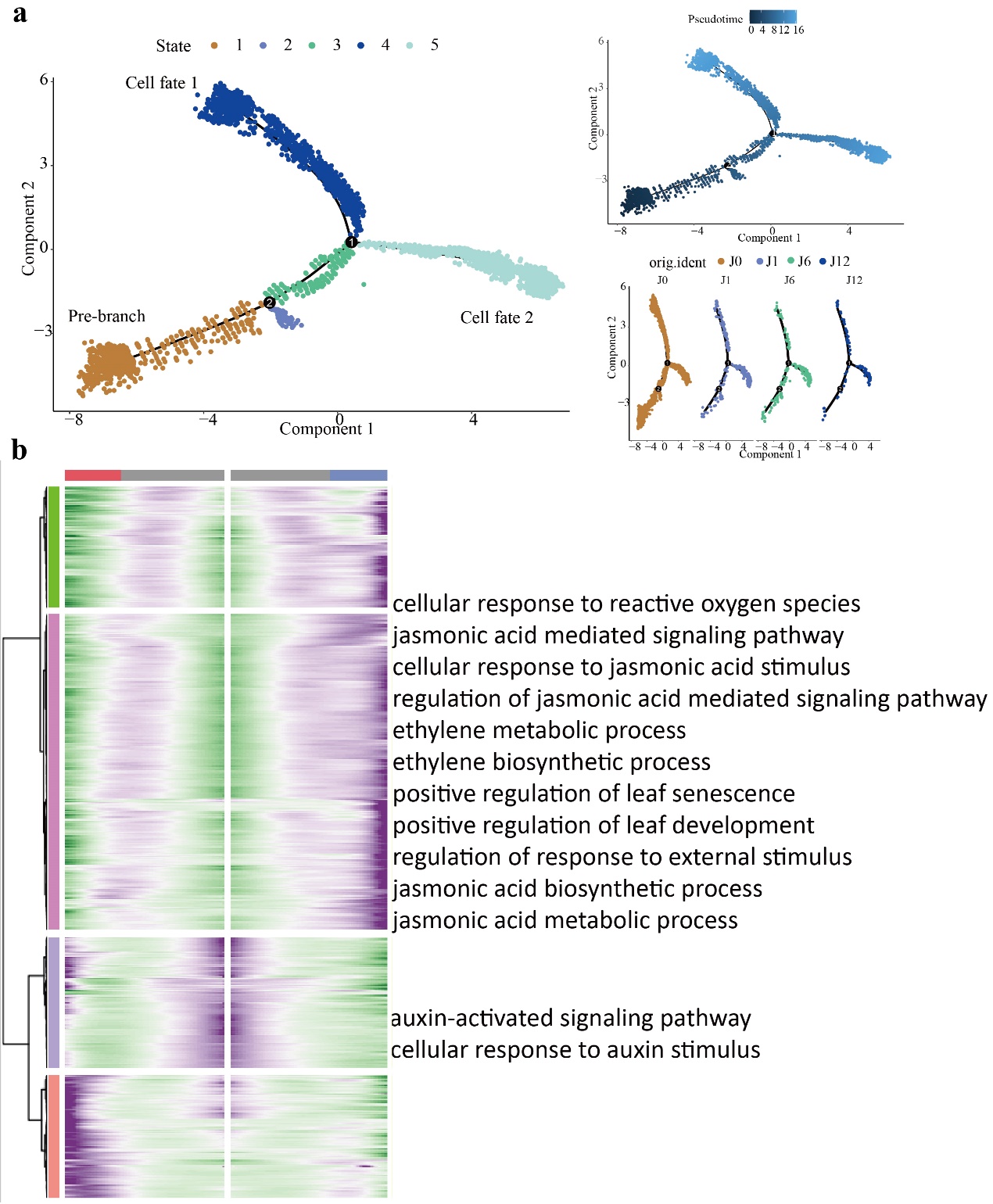


**Fig. S6. Pseudotime trajectory of primary xylem of Jin668. (a)** Pseudotime analysis of primary xylem of Jin668 with induction time. Each dot indicates a single cell, and the color of the upper right corner represents the starting point and end point of differentiation. **(b)** Heatmap showing the expression of the branch-dependent genes over pseudo-time. GO terms are shown in the table on the right. The middle of the heatmap is the beginning of pseudo-time. Both sides of the heatmap are the end of pseudo-time. Color bar indicates the relative expression level.


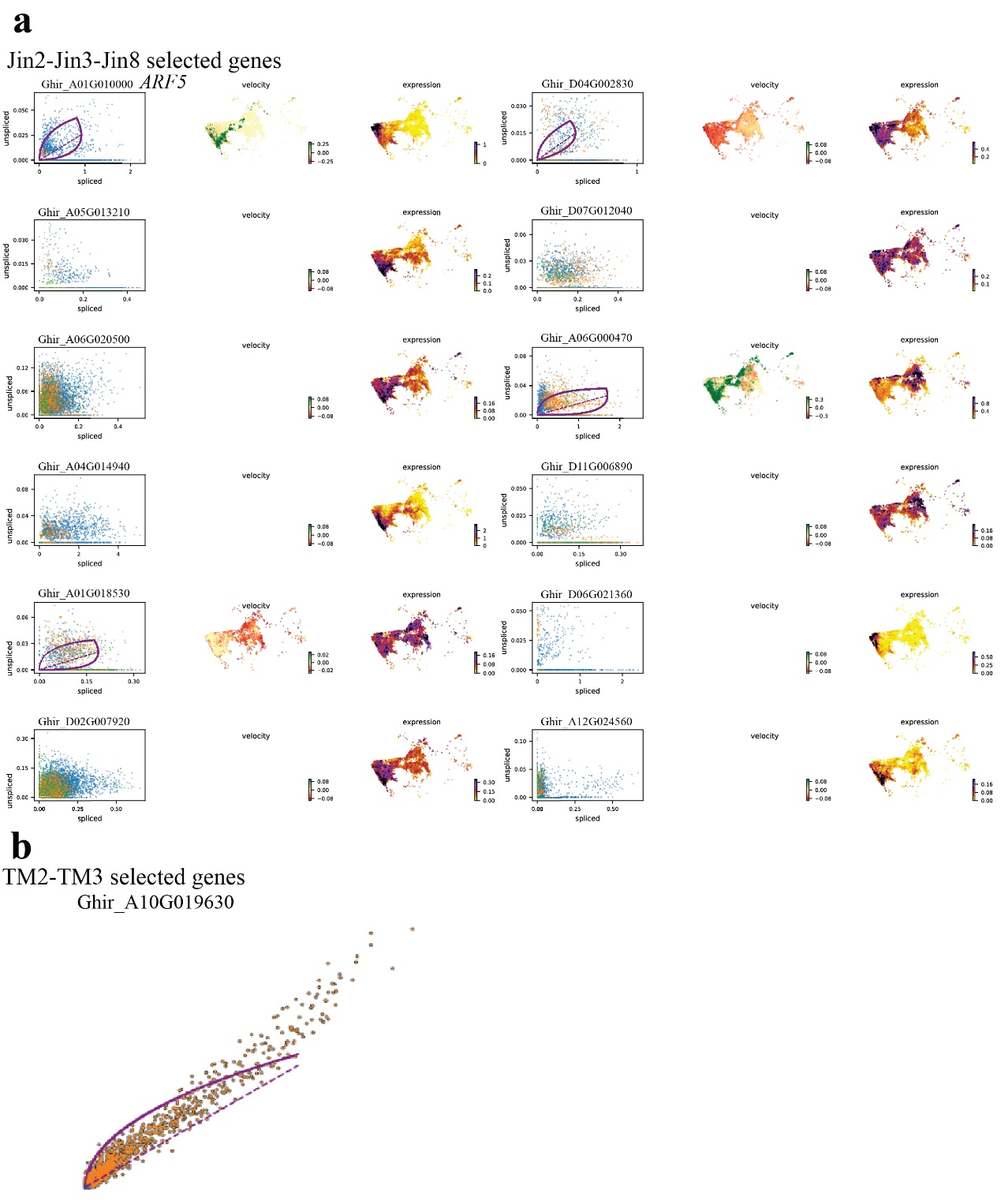


**Fig. S7. The fate maps of directional aggregation and gene expression trend of local cell clusters. (a)** SE related genes in RNA velocity of primary xylem, parenchyma cells and cambium region of Jin668. **(b)** SE related genes in RNA velocity of primary xylem, parenchyma cells of TM-1. Fate maps, phase portraits, unspliced residuals and smoothed gene expression trends are shown from left to right for these driver regulated genes.
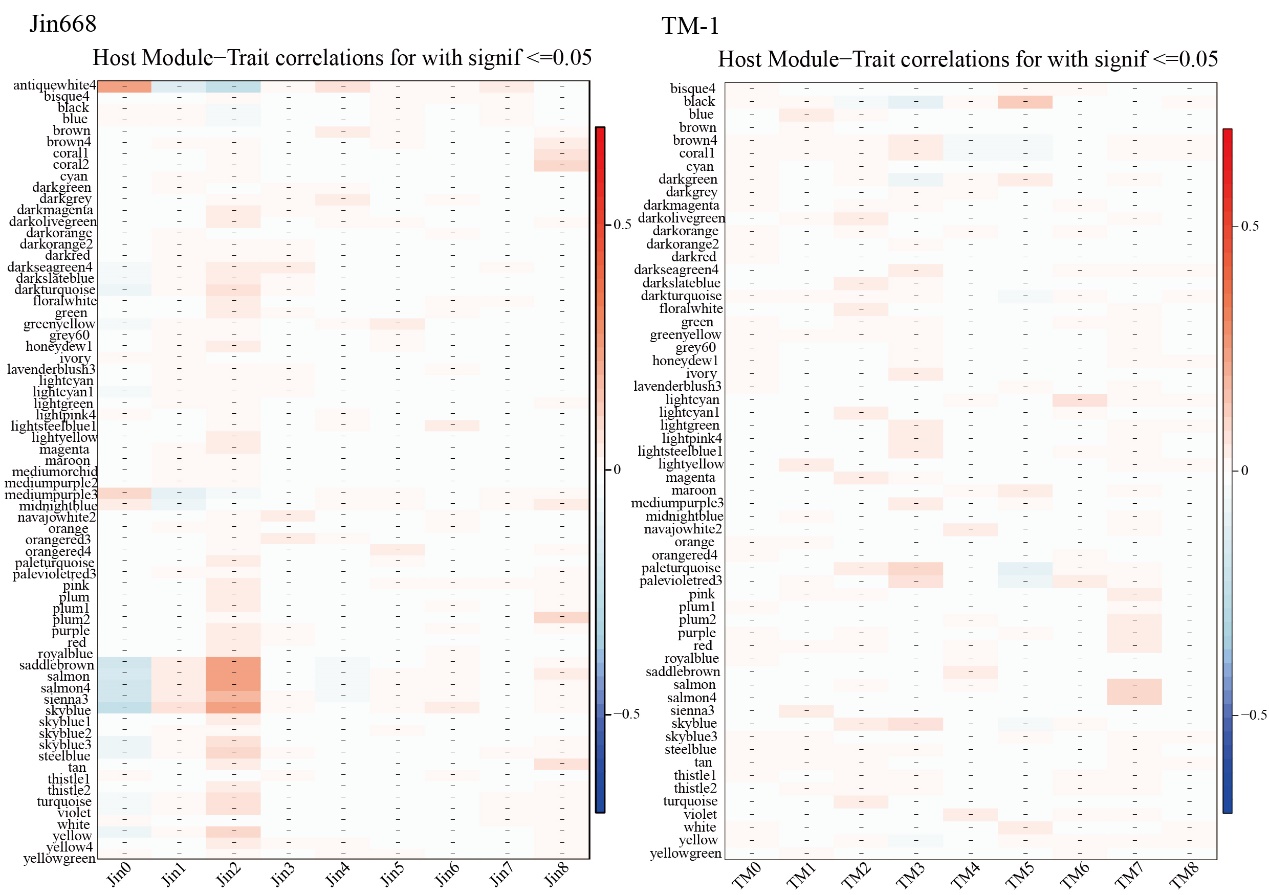


**Fig. S8. Network analysis SE-related genes of Jin668 and TM-1 hypocotyl primary vascular cells.** According to Pearson’s correlation analysis, the correlations between color modules and each samples in Jin668 and TM-1 were revealed. Red represents positive correlation and blue represents negative correlation.


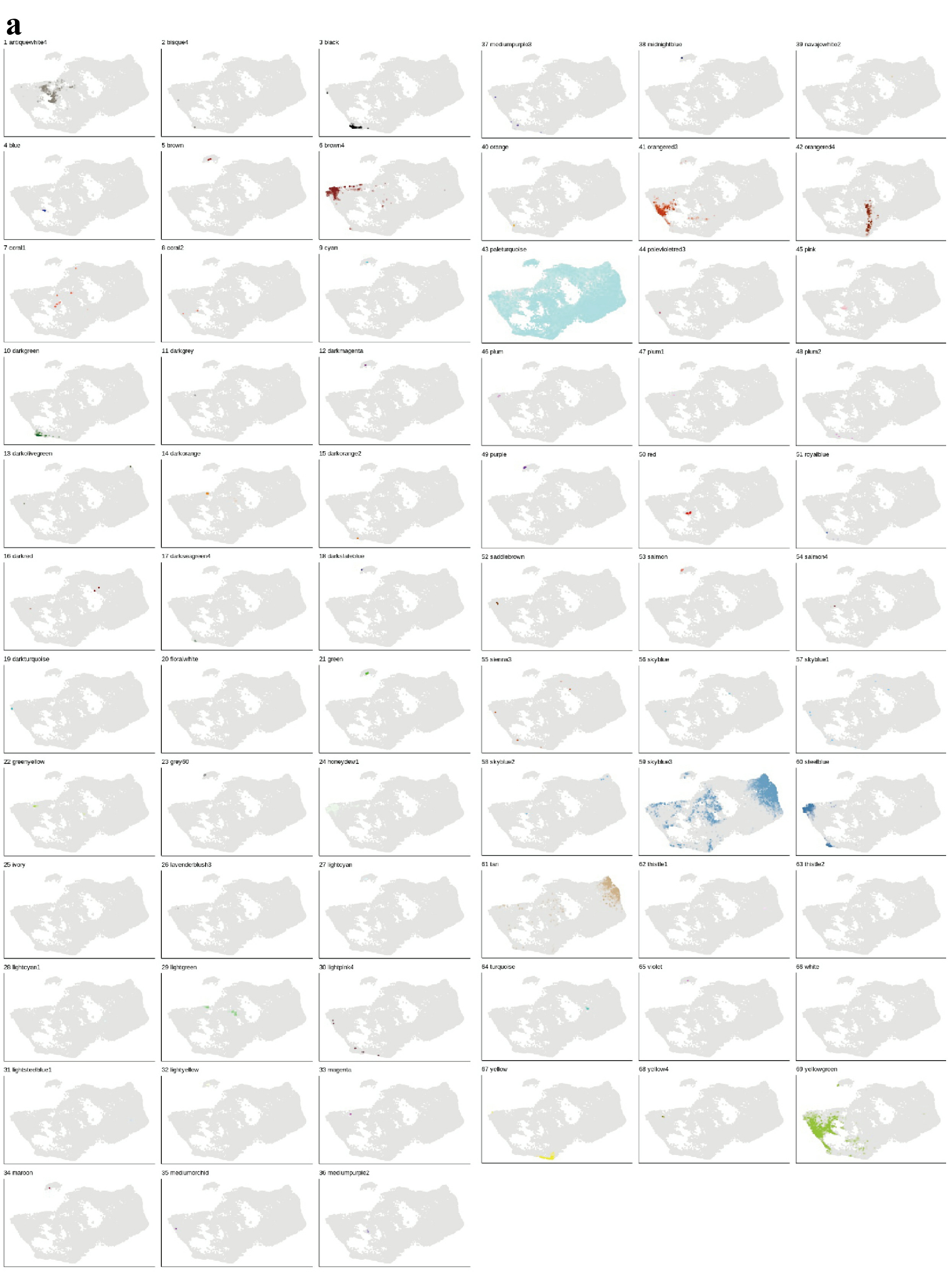

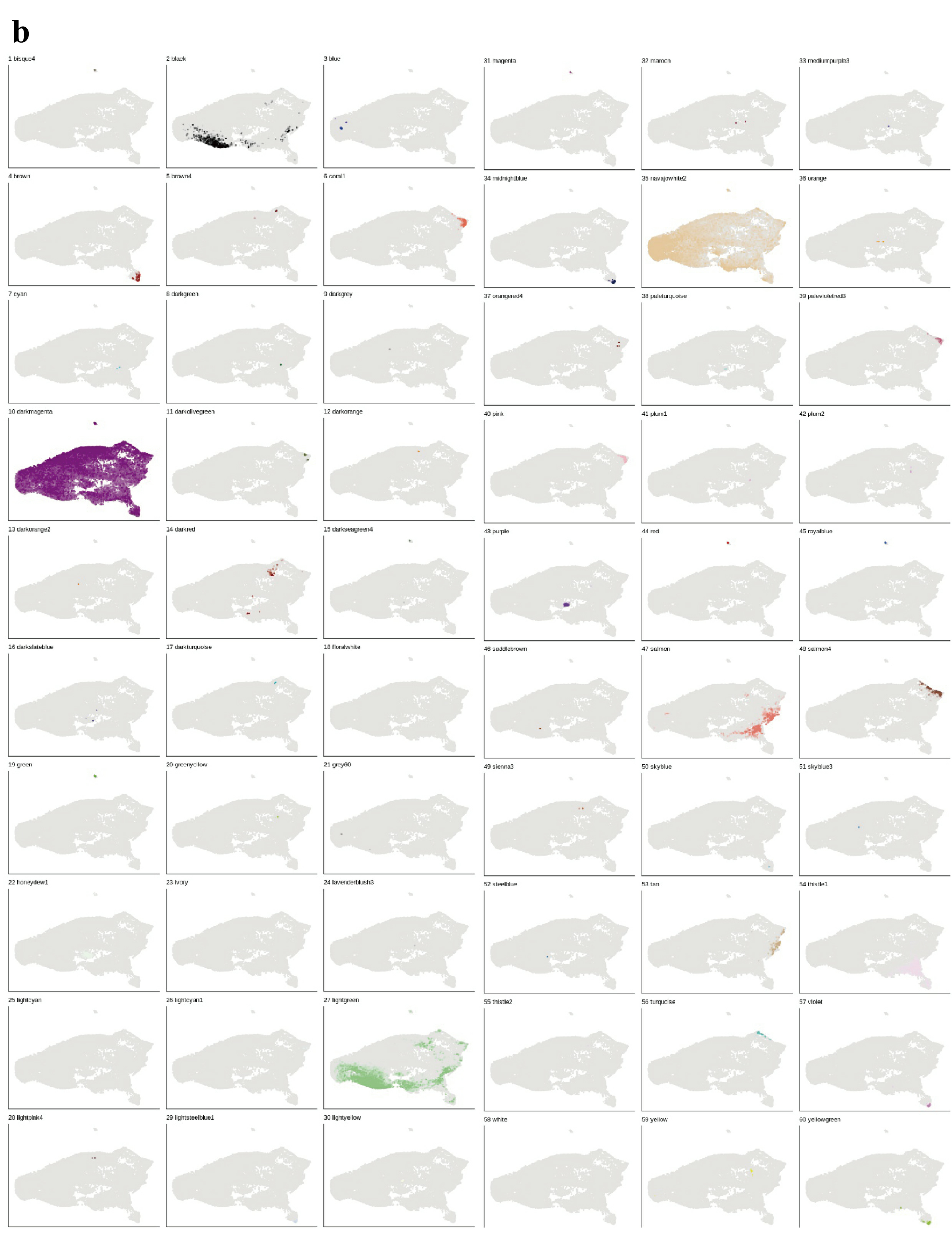


**Fig. S9. Gene regulation and expression modules in different cell types. (a)** 69 regulatory Modules of different cell clusters in Jin668. **(b)** 60 regulatory Modules of different cell clusters in TM-1. The color represents the cell cluster where the regulatory modules were located.


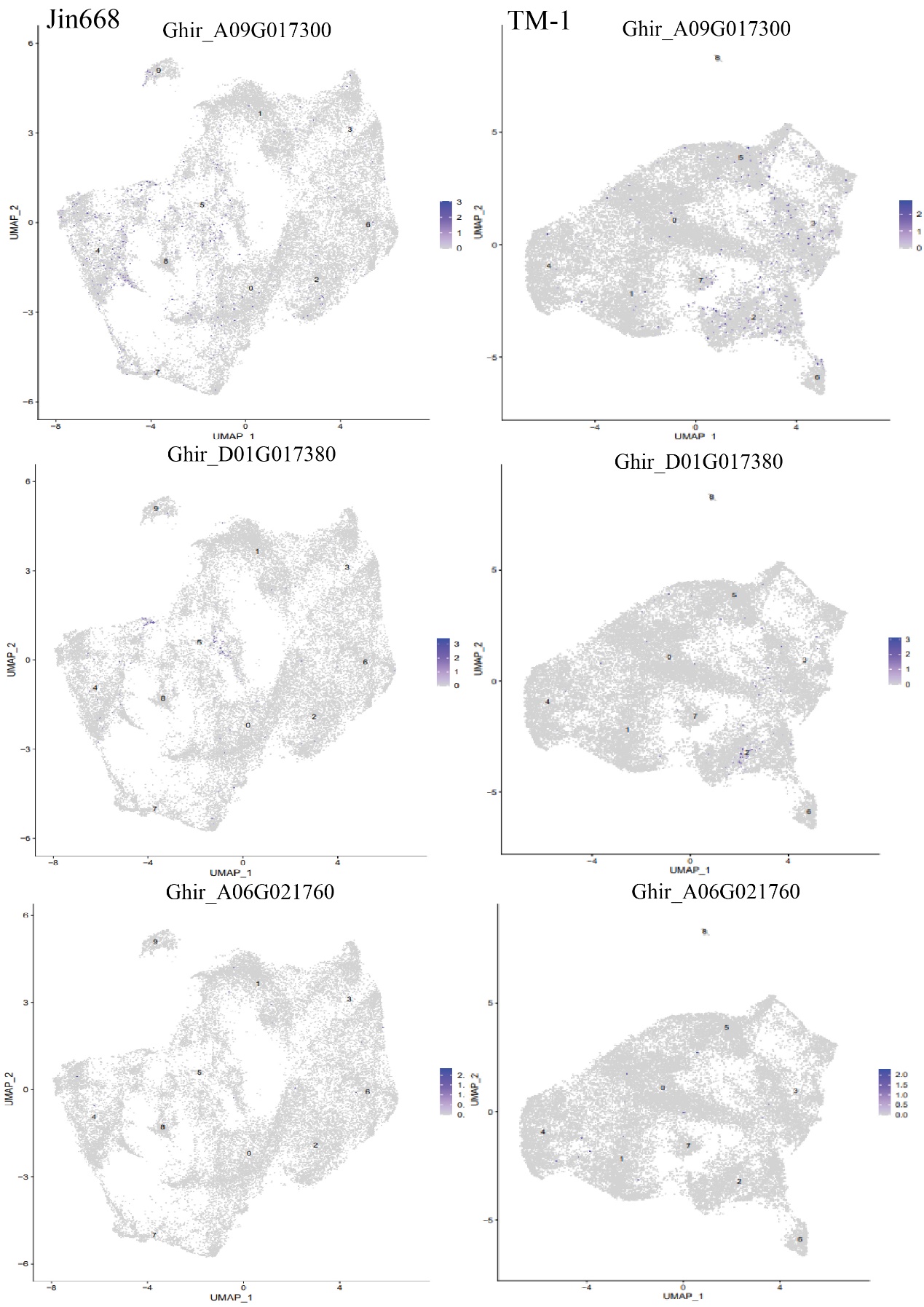


**Fig. S10. The expression patterns of selected SE related genes in Jin668 (left) and TM-1 (right).**


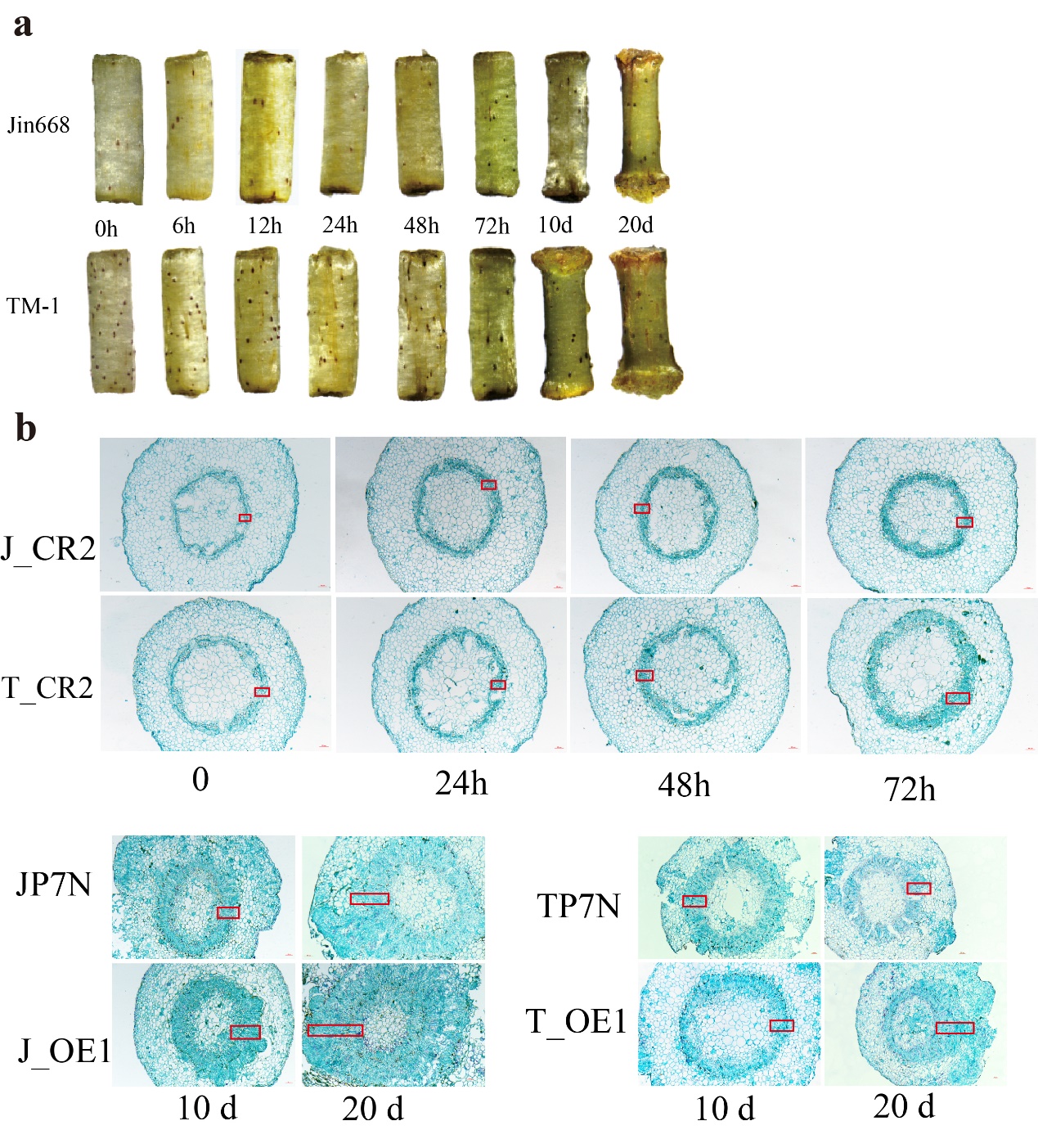

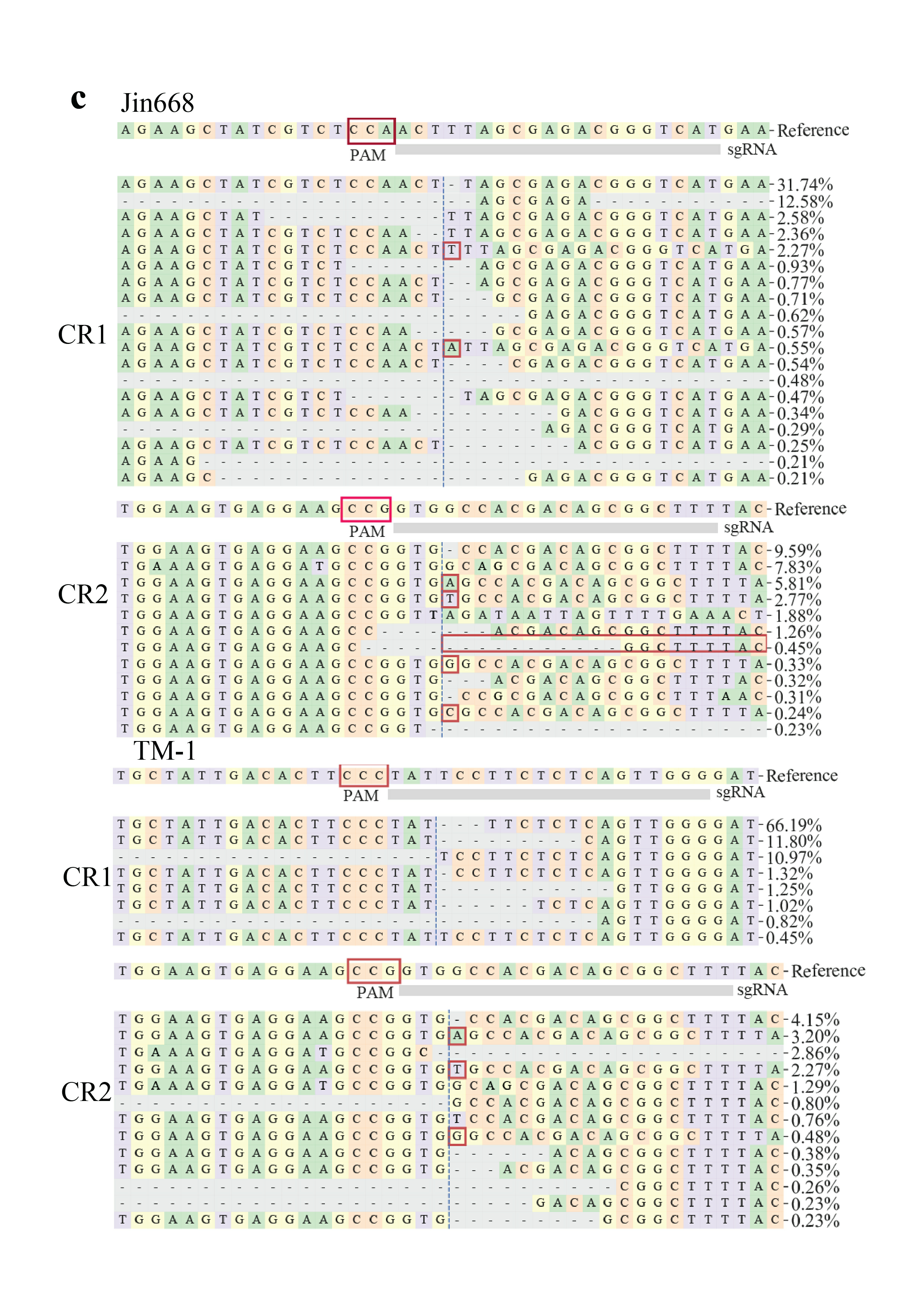


**Fig. S11.** **Phenotype of knock out with hypocotyls as explants. (a)** Morphological changes of hypocotyls of Jin668 and TM-1 induced at different times (From 0 to 20d). **(b)** Paraffin sections of hypocotyls of J_CR2 and T_CR2 after induction on callus induction medium for 0, 24, 48, 72 hours. JP7N and JOE1 after induction on callus induction medium for 10 days, 20days. The red box represents the proliferation site. Scale bar, 100 μm. **(c)** Mutant genotype of CR1 and CR2 of Jin668 and TM-1.
