# Supplementary material for: Single-cell resolution analysis reveals the preparation for reprogramming the fate of stem cell niche in cotton lateral meristem
Source: Genome Biol. 2023 Aug 25;24:194. doi: 10.1186/s13059-023-03032-6 (PMC10463415; doi:10.1186/s13059-023-03032-6)
Supplement: Supplementary file 6 — Additional file 6. Review history. [file 13059_2023_3032_MOESM6_ESM.docx]

Review History

**First round of review**

**Reviewer 1**

**Were you able to assess all statistics in the manuscript, including the appropriateness of statistical tests used?**

No, I do not feel adequately qualified to assess the statistics.

**Comments to author:**

The manuscript entitled "Reprogramming of the Fate of Plant Stem Cell Niches in lateral meristems enables the cells plant regeneration ability as revealed at Single-cell resolution" by Zhu et al. reported the scRNA-seq analysis of somatic embryogenesis in cotton and identified several key genes involved in this process. There are two major findings of the manuscript. First, the authors provided a single cell transcriptome tool. Second, the authors indicated that some key genes are responsible for the regeneration efficiency difference in Jin668 and TM-1. As a whole, the data are potentially interesting and might be a useful tool in the field of cotton regeneration. I have several suggestions for the authors to improve the manuscript.

Major points:

1) The authors performed scRNA-seq analysis using explants at 0, 1, 6, 12 h of tissue culture. However, the cell division seems to be much later than the time points of scRNA-seq. What is the cell division phenotype of explants at 12 h? Are the cells entering S or M phase? Therefore, I am not sure that the scRNA-seq time points used by the authors could be related to cell reprogramming (the time points might reflect the preparation for cell reprogramming). I think the authors should carefully revise the interpretation (including the title).

2) More experimental (genetic, in situ, or others) validations should be included. For examples, more cell clusters in UMAP should be validated, as well as the data in pseudotime and WGCNA.

3) The data of LAX2 are important in this study. The authors nicely showed the phenotype of callus formation, but I would like to suggest the authors add some phenotype analysis of somatic embryogenesis.

Minor points:

1) The genes annotation listed in transcriptome analysis are not consistent with the text. For examples, lines 179-230. It is difficult to follow the gene names and their numbers in the figure.

2) Figure 2E, tissue structures and cell types should be annotated.

**Reviewer 2**

**Were you able to assess all statistics in the manuscript, including the appropriateness of statistical tests used?**

Yes, and I have assessed the statistics in my report.

**Comments to author:**

In their work "Reprogramming of the Fate of Plant Stem Cell Niches in lateral meristems enables the cells plant regeneration ability as revealed at Single-cell resolution", Zhu et al. profiled the single-cell transcriptomes of hypocotyl tissue in two genotypes of upland cotton Gossypium hirsutum, Jin668 and TM-1, which have different abilities of somatic embryogenesis and regeneration (regenerable vs. recalcitrant) and suggested that the primary vascular tissue is the major cell type undergoing cell fate transition in somatic embryogenesis (SE). Overall this work could be of general interest to the field of plant regeneration. However, I do have some specific concerns about this manuscript.

1. What are the genomic differences between the two genotypes Jin668 and TM-1? There does not seem to be much text in Introduction or Discussion that addresses this aspect. Do Jin668 and TM-1 also differ in their single-cell transcriptomic profiles of hypocotyl tissue without induction for SE? How do we know if their scRNA-seq cluster differences stem from their differences in SE rather than other genomic differences unrelated to SE?

2. The study used RNA velocity to analyze the fate determination of primary vascular cells (Fig. 6). As pointed out in the text (line 388), RNA velocity is inferred from splicing variants of transcripts. How much of such splicing variation can be profiled by the 10x Genomics scRNA-seq platform used in this study? From what I understand, the 10x Genomics platform constructs libraries where transcripts are only sequenced at one of the two ends. In that case, very little, if any, information on splicing can be extracted from the sequencing data.

3. The relationships between Jin668 and TM-1 cell clusters also seem to be quite confusing. In Figure 2 B and C, we have different cell types labeled with the same colors in the two genotypes and same cell types labeled with different colors. What kinds of analyses and experimental evidence actually support these correspondence relationships between cell clusters of the two genotypes? This would be critical to the conclusions drawn in other parts of the manuscript.

**Authors Response**

**Point-by-point responses to the reviewers’ comments:**

Reviewer #1:

We sincerely appreciate the editors and reviewers for their thoughtful comments and recommendations on our manuscript. Those comments are very helpful for revising and improving our paper, as well as the important guidance to other research. We have studied the comments carefully and have modified the manuscript accordingly. We hope this revised manuscript will meet the journal’s high standards. The main corrections are included in the revised manuscript and the response to the reviewers’ comments are as follows:

The manuscript entitled "Reprogramming of the Fate of Plant Stem Cell Niches in lateral meristems enables the cells plant regeneration ability as revealed at Single-cell resolution" by Zhu et al. reported the scRNA-seq analysis of somatic embryogenesis in cotton and identified several key genes involved in this process. There are two major findings of the manuscript. First, the authors provided a single cell transcriptome tool. Second, the authors indicated that some key genes are responsible for the regeneration efficiency difference in Jin668 and TM-1. As a whole, the data are potentially interesting and might be a useful tool in the field of cotton regeneration. I have several suggestions for the authors to improve the manuscript.

Major points:
1) The authors performed scRNA-seq analysis using explants at 0, 1, 6, 12 h of tissue culture. However, the cell division seems to be much later than the time points of scRNA-seq. What is the cell division phenotype of explants at 12 h? Are the cells entering S or M phase? Therefore, I am not sure that the scRNA-seq time points used by the authors could be related to cell reprogramming (the time points might reflect the preparation for cell reprogramming). I think the authors should carefully revise the interpretation (including the title).

***Response:*** *Thanks for your valuable comments of this manuscript. We conducted continuous sectioning during the early stage of hypocotyl induction, including 0h (0 hours after induction), 6h, 12h, 24h, and 48h. As shown in the figure, the proliferation of primary vascular tissue of hypocotyl was observed from 12 hours.* *Therefore, we think that the cells of the hypocotyl, at least the cells of the primary vascular tissue, have completed at least one cell cycle activity. Likewise, we speculate that genes related to callus proliferation may have been expressed before induction for 12 hours. Callus formation is the main stage of somatic embryogenesis. We want to know whether there is a kind of cells in renewable materials that respond quickly to the process of cell reprogramming and are easier to dedifferentiate into callus, thus forming somatic cell embryos (Figure 1).*

renewable materials that respond quickly to the process of cell Reprogramming and are easier to dedifferentiate into callus, thus forming somatic embryos.

Figure 1. Paraffin sections of cotton hypocotyls at different induction times. Scale bars represent 100μm.

**Question** 2) More experimental (genetic, in situ, or others) validations should be included. For examples, more cell clusters in UMAP should be validated, as well as the data in pseudotime and WGCNA.

***Response:*** *Thanks for your valuable comments of this manuscript. As suggestion, we performed RNA in situ hybridization experiments to verify all of the cell types in the UMAP map. In addition, the LAX2 gene that was selected to perform CRISPR and overexpression assay was also can be tracked in pseudotime and WGCNA.*
**Question** 3) The data of LAX2 are important in this study. The authors nicely showed the phenotype of callus formation, but I would like to suggest the authors add some phenotype analysis of somatic embryogenesis.
***Response:*** *Thanks for your valuable comments of this manuscript. As suggested, we added the somatic embryogenesis phenotype of LAX2 gene, including the time of somatic embryogenesis and morphological analysis of somatic embryos. Overall, overexpression* *of LAX2 (OE1) improved the regeneration efficiency of Jin668. The time for the formation of embryogenic callus in transgenic materials is about 70 days, 20 days earlier than the control. The induced hypocotyls can basically form embryogenic callus tissue, increasing the number of somatic embryos. Many hypocotyls directly induce yellow and green embryoids on 2,4-D induction medium. In differentiation medium, the transgenic material can form normal somatic cell embryos, including globular embryo, heart-shaped embryos, torpedo embryos and cotyledon embryos, and finally form complete transgenic plants (Figure 2).*

Figure 2. Observation of somatic Embryogenesis Phenotype of OE1. Scale bars represent 10 mm.

**Question** Minor points:
1) The genes annotation listed in transcriptome analysis are not consistent with the text. For examples, lines 179-230. It is difficult to follow the gene names and their numbers in the figure.

2) Figure 2E, tissue structures and cell types should be annotated.
***Response:*** *Thanks, as suggested, we have rectified the genes annotation throughout the manuscript and annotated the tissue structures and cell types in Figure 2E.*

**Reviewer #2:**

In their work "Reprogramming of the Fate of Plant Stem Cell Niches in lateral meristems enables the cells plant regeneration ability as revealed at Single-cell resolution", Zhu et al. profiled the single-cell transcriptomes of hypocotyl tissue in two genotypes of upland cotton Gossypium hirsutum, Jin668 and TM-1, which have different abilities of somatic embryogenesis and regeneration (regenerable vs. recalcitrant) and suggested that the primary vascular tissue is the major cell type undergoing cell fate transition in somatic embryogenesis (SE). Overall this work could be of general interest to the field of plant regeneration. However, I do have some specific concerns about this manuscript.
**Question** 1. What are the genomic differences between the two genotypes Jin668 and TM-1? There does not seem to be much text in Introduction or Discussion that addresses this aspect. Do Jin668 and TM-1 also differ in their single-cell transcriptomic profiles of hypocotyl tissue without induction for SE? How do we know if their scRNA-seq cluster differences stem from their differences in SE rather than other genomic differences unrelated to SE?
***Response:*** *We thank the reviewer for this useful comment. The Jin668 is a Upland cotton genotypes with high regeneration efficiency, while the TM-1 is a genetic and genomic standard line for Upland cotton with no-regeneration ability. Currently, multiple versions* *of high-quality genome sequencing for TM-1 have been completed (Wang et al., 2019). Recently, we have also conducted a genome sequencing for the Jin668 genotype, which get a T2T-grade genome with scaffolds N50 of 108.10 Mbp. Subsequently, comparative genomic analysis of Jin668 and TM-1 showed approximately 89.4% similarity in their genomic sequences. This part of the work is currently in progress, so we did not present it in this manuscript.*

*Jin668 and TM-1 have a similar regeneration process and both can induce callus formation, especially in Jin668, where callus can further differentiate into a loose, bright yellow embryonic callus (EC), while in TM-1, the callus appears as a compact, globular and green colored non-embryogenic callus (NEC), which is incapable of forming EC and further induce embryoids (Figure 3).*

Figure 3. The SE-based regeneration process and corresponding time points (unit: day) of Jin668 and TM-1. Scale bars represent 1 mm.

*Considering the high degree of similarity in genomic sequence and induction process during the pre-regeneration stage of both Jin668 and TM-1 genotypes, as well as their comparable single-cell transcriptomic profiles (Fig. 3A) at the time of induction, we undoubtedly believe that there will inevitably be SE related and unrelated differences due to genotype differences between them. Nonetheless, we mainly rely on previous research to select SE related genes and investigate their expression differences at different induction times for both genotypes.*

**Question** 2. The study used RNA velocity to analyze the fate determination of primary vascular cells (Fig. 6). As pointed out in the text (line 388), RNA velocity is inferred from splicing variants of transcripts. How much of such splicing variation can be profiled by the 10x Genomics scRNA-seq platform used in this study? From what I understand, the 10x Genomics platform constructs libraries where transcripts are only sequenced at one of the two ends. In that case, very little, if any, information on splicing can be extracted from the sequencing data.
***Response:*** *Thanks. These are helpful comments. In our study, we analyzed the RNA velocity based on the official guide of 10x Genomics (*[*https://www.10xgenomics.com/resources/analysis-guides/trajectory-analysis-using-10x-Genomics-single-cell-gene-expression-data*](https://www.10xgenomics.com/resources/analysis-guides/trajectory-analysis-using-10x-Genomics-single-cell-gene-expression-data)*). As suggestion, we have checked these splicing variation and detected an average of 8% unspliced RNA in the cell clusters of Jin668 and TM-1 genotypes (Figure 4).*

**Question** 3. The relationships between Jin668 and TM-1 cell clusters also seem to be quite confusing. In Figure 2 B and C, we have different cell types labeled with the same colors in the two genotypes and same cell types labeled with different colors. What kinds of analyses and experimental evidence actually support these correspondence relationships between cell clusters of the two genotypes? This would be critical to the conclusions drawn in other parts of the manuscript.

***Response:*** *Thanks for your valuable comments of this manuscript. We have made modifications to Figures 2B and 2C, where the same color represents the same cell types. Among them, the cambium cells in Jin668 and the mature phloem cells in TM-1 were labeled with different colors. As mentioned above (****Question*** ***1****), the genotypes of Jin668 and TM-1 have highly similar* *genomic sequences and hypocotyl regeneration processes. Therefore, for the correspondence* *between cell types in the two materials, we initially assumed that the cell types in both hypocotyls were largely consistent; Then, based on the expression of marker genes in different cell clusters, we identified the cell clusters expressing the same marker genes as the same cell types. According to the comments, we selected marker genes that were expressed in both varieties for RNA in situ hybridization, and the experimental results showed that these cell types were indeed consistent (Figure 5).*

Figure 5. RNA in situ hybridization of marker genes both expressed in Jin668 and TM-1. The gene expression site wrer indicated by red arrow. Scale bars represent 50 μm.

*Wang, M., Tu, L., Yuan, D., Zhu, D., Shen, C., Li, J., Liu, F., Pei, L., Wang, P., Zhao, G., et al. (2019). Reference genome sequences of two cultivated allotetraploid cottons, Gossypium hirsutum and Gossypium barbadense. Nature Genetics 51, 224-229.*

**Second round of review**

**Reviewer 1**

The authors have addressed many of my concerns. I have no further suggestions.

**Reviewer 2**

The authors have addressed my comments. In the revised title, maybe it should be "the preparation for" instead of "the preparation of".
